# Angiopoietin-like protein 8 orchestrates macrophage glycogen metabolism and polarization via the JNK signaling pathway in cytokine storm syndrome

**DOI:** 10.1186/s13578-025-01487-7

**Published:** 2025-10-15

**Authors:** Yang Su, Rongtian Zhang, Kongdong Li, Hong Shen, Mengjiao Nan, Chang Liu, Wenxiang Zhang, Siyu Chen

**Affiliations:** State Key Laboratory of Natural Medicines, School of Life Science and Technology, China Pharmaceutical University, Nanjing, 211198 China

**Keywords:** Cytokine storm syndrome, Angptl8, Macrophage polarization, Glycogen metabolism, Liver

## Abstract

Cytokine storm syndrome (CSS) is associated with severe damage and high mortality in acute diseases. Over-activation of M1 macrophages, accompanied with excessive pro-inflammatory cytokine secretion, drives cytokine storms, while promoting M2 macrophage polarization is a potential CSS treatment. The liver, an immune-responsive organ, secretes hepatokines such as fibroblast growth factor-21 (FGF-21) to regulate macrophage activation, but knowledge of their role in CSS-related inflammation is elusive, fueling the search for new hepatokines that can effectively fine-tune the pro-inflammatory activation of macrophages during CSS. In this study, lipopolysaccharide (LPS)-induced CSS signals increase hepatic Angiopoietin-like protein 8 (Angptl8) expression. Angptl8 knockout (Angptl8^−/−^) reduces mortality in high-dose LPS-treated mice. This is due to inhibited M1 and enhanced M2 macrophage polarization, decreased pro-inflammatory cytokines, and alleviated CSS symptoms. Angptl8 promotes M1 polarization by activating glycogen metabolism via c-Jun N-terminal kinase (JNK) phosphorylation. Mice treated with an Angptl8-neutralizing antibody have improved CSS symptoms, and the antibody is non-toxic in vivo. Hence, Angptl8 is a promising CSS therapeutic target. Given cytokine storms’ role in viral infections and immune therapy-related adverse reactions, targeting Angptl8 may provide new treatments, potentially improving patient outcomes and reducing morbidity and mortality.

**Supplementary Information:**

The online version contains supplementary material available at 10.1186/s13578-025-01487-7.

## Introduction

Cytokine storm syndrome (CSS) is a life-threatening condition characterized by severe systemic inflammation [1, 2]. It results from an exaggerated immune response of the innate immune system, which can be triggered by various stimuli, including therapeutic measures, microbial infections, and autoimmune diseases [3]. High mortality rates, reaching up to 25%, have been documented, especially during the COVID-19 pandemic [4, 5]. Despite considerable progress in elucidating its pathophysiology and developing therapeutic interventions, patients with CSS continue to face formidable survival challenges. In clinical practice, the management of CSS often requires a multifaceted approach, encompassing antibacterial and anti-inflammatory strategies, including the administration of antibiotics, hormonal medications to regulate inflammation, fluid resuscitation, vasopressin therapy, mechanical ventilation, and even organ replacement [6, 7]. However, despite technological advancements enabling complex interventions in CSS, there continues to be a risk of complications arising from improper management, particularly those associated with the excessive use of antibiotics and hormones, as well as injuries stemming from respiratory interventions [8]. Consequently, there is an urgent need for the exploration of novel therapeutic strategies for CSS, necessitating the development of safer and more efficacious treatments to enhance patient outcomes.

During immune defense and elimination of invading pathogens, macrophages may generate an excessive amount of inflammatory cytokines, triggering an inflammatory storm that causes indiscriminately damage to normal tissues, ultimately resulting in organ failure and increased mortality [9, 10]. Consequently, establishing a precise equilibrium between pro-inflammatory (M1) and anti-inflammatory (M2) macrophage activities, while concurrently suppressing the macrophage-mediated excessive inflammatory response, is crucial for maintaining normal organ function during CSS, decreasing CSS-associated mortality, and enhancing patient outcomes [11]. Current therapeutic interventions, such as the administration of glucocorticoids for their broad-spectrum anti-inflammatory effects or cytokine-directed therapy, are designed to mitigate excessive immune responses. Nevertheless, these methodologies may inadvertently elicit adverse effects, such as hypertension and damage to organs, particularly impacting the liver, which functions as a pivotal defense against external pathogens [12].

Due to its anatomical characteristics and distinctive circulatory architecture, the liver serves as the primary defense barrier of the body, ingesting and eliminating invading bacteria and their metabolites [13]. Upon activation of the innate immune defense mechanism, which comprises endothelial cells, neutrophils, and hepatic macrophages (Kupffer cells and monocyte-derived macrophages), the liver transitions from its metabolic role to immunogenic responses. This transition facilitates the generation and secretion of acute phase proteins, allowing the liver to adapt to the infectious environment [13–16]. Recent studies have suggested the potential of targeting the liver as a protective mechanism against CSS, however, there is still a lack of effective strategies to alleviate the harmful effects of this severe complication. Furthermore, the intricate mechanisms underlying the liver-dependent activation of macrophages during CSS have not yet been fully elucidated. It is noteworthy that, as an immune-responsive organ, the liver demonstrates a sensitive reaction to inflammatory signals and secretes hepatokines, which serve to regulate the activation of macrophages. A representative instance of this is fibroblast growth factor-21 (FGF-21), a metabolically active factor produced by hepatocytes. Its expression is upregulated in hepatocytes and macrophages stimulated by lipopolysaccharide (LPS). Additionally, PF-05231023, a long-acting analog of FGF-21, has demonstrated protective effects against septic liver injury by inhibiting pro-inflammatory macrophage activation and enhancing autophagic degradation of hypoxia-inducible factor-1α (HIF-1α) [8]. However, the limited circulating half-life of recombinant FGF-21 constrains its potential for treating CSS. These questions prompt us to discover novel hepatokines that can effectively modulate the pro-inflammatory activation of macrophages in response to CSS.

Angiopoietin-like 8 (Angptl8), a member of the Angptl protein family, distinguishes itself from Angptl1-7 due to its unique characteristics, which include the absence of common features such as the N-terminal coiled-coil domain, C-terminal fibrinogen-like domain, and glycosylation patterns [17]. Angptl8 exhibits high expression levels in the liver and brown adipose tissue of mice, while in humans, it is exclusively expressed in the liver, indicating its role in hepatic secretion [18, 19]. The Angptl8 protein serves a dual function, either directly or indirectly promoting the cleavage of Angptl3, thereby influencing the activity of lipoprotein lipase (LPL) and modulating lipid metabolism [20]. This relationship highlights Angptl8’s crucial link to the pathogenesis of various metabolic diseases, including dyslipidemia, non-alcoholic fatty liver disease, and type 2 diabetes. Furthermore, our previous studies have revealed that Angptl8 binds to its receptor, leukocyte immunoglobulin-like receptor B2 (LILRB2), also called paired immunoglobulin-like receptor (PirB), and orchestrates the food-induced resetting of the hepatic circadian clock in mice, hinting at its potential as a novel *Zeitgeber* for peripheral clocks [21]. Additionally, a correlation has been observed between elevated plasma Angptl8 levels and the inflammation response syndrome, with TNF-α and LPS stimulation known to induce liver expression of this protein [22]. Notably, deficiency of Angptl8 has been shown to ameliorate LPS-induced liver injury by improving lipid metabolic imbalances [23]. Importantly, the receptor for Angptl8, LILRB2/PIRB, plays a pivotal role in macrophage recruitment during the fibrogenic progression of non-alcoholic steatohepatitis [24, 25]. These findings emphasize the potential involvement of Angptl8 in inflammatory regulation. However, the definitive role of Angptl8 in inflammation and the optimal therapeutic targeting in LPS-induced CSS remain areas requiring further investigation.

In this study, we observed an increase in the expression of hepatic Angptl8 in response to LPS-induced CSS signals. Notably, the absence of Angptl8 in mice subjected to high-dose LPS treatment led to a reduction in mortality. This protective effect is attributed to the inhibition of M1 macrophage polarization and the enhancement of M2 polarization, resulting in decreased levels of pro-inflammatory cytokines and alleviated consequences of LPS-induced CSS. Mechanistically, Angptl8 facilitates the polarization of macrophages towards the M1 phenotype via the activation of glycogen metabolism, in a process that is dependent upon the phosphorylation of the c-Jun N-terminal kinase (JNK) protein. Additionally, noteworthy improvements in LPS-induced CSS symptoms was observed in mice administrated with an Angptl8-neutralizing antibody. Additionally, this neutralizing antibody exhibited no toxicity in in vivo tests. Collectively, our findings validate that Angptl8 represents a promising target for the treatment of CSS, potentially providing therapeutic advantages for patients, particularly those affected by viral infections and immune therapies.

## Materials and methods

### Animals

Male WT mice and Angptl8 knockout (Angptl8^−/−^) mice in a C57BL/6J background were purchased from the GemPharmatech (Nanjing, China). All animal procedures in this investigation were conducted in accordance with the Guide for the Care and Use of Laboratory Animals published by the US National Institutes of Health (NIH publication No. 85-23, revised 1996) and the approved regulations set by the Laboratory Animal Care Committee at China Pharmaceutical University (Permit number SYXK-2021-0011). All mice were maintained in a 12 h light/dark cycle and in a temperature- and humidity-controlled environment. To establish the CSS mouse model, 8-week-old mice were injected intraperitoneally (*i*.*p*.) with 25 mg/kg LPS (Beyotime, Nanjing, Jiangsu, China) [11, 26]. To investigate the role of Angptl8 in LPS-induced CSS, Angptl8^−/−^ mice were challenged with 25 mg/kg LPS (*i.p.*) for 6 h, and the mice were sacrificed to collect tissues and sera. Additionally, the immuno-neutralized Angptl8 antibody (designed according to a previous study and synthesized by Bioworld Corp., Nanjing, China, at doses of 10 µg/25 g and 50 µg/25 g of mouse body weight) or control IgG was intravenously (*i.v.*) injected into mice 2 h before LPS challenged to quench Angptl8 [21, 27].

### Cell culture

PHs and kuffer cells were isolated from mice using the collagenase IV (Sigma, Shanghai, China) perfusion method as previously described and cultured in a humidified atmosphere containing 5% CO_2_ at 37 °C [28, 29]. For the preparation of bone marrow-derived macrophages (BMDMs), femurs were aseptically harvested from mice. Bone marrow cells were flushed from the bones and cultured in RPMI 1640 media supplemented with 20 ng/mL macrophage colony-stimulating factor (M-CSF, ABclonal Biotechnology, Wuhan, China). The medium was refreshed every two days, and the cells differentiated into BMDMs within one week. For M1-like activation, BMDMs were treated with 100 ng/mL LPS for 12 h. For M2 polarization, cells were treated with 20 ng/mL interleukin-4 (IL-4; Peprotech, USA) for 24 h. Recombinant mouse His-Angptl8 protein (Cloud-Clone Corp., Wuhan, China) was added 1 h before IL-4 stimulation. All cells were maintained in a humidified incubator with 5% CO_2_ at 37 °C.

### Flow cytometry analysis

Cells were collected on a BD FACSFortessa instrument (BD Biosciences, CA, USA) and analyzed in FlowJo10 software. Fluorescent labeled antibodies, including purified anti-mouse CD16/32 (Biolegend, Cat. No. 101319, 1 µg/test), ABflo^®^ 594 Rabbit anti-Mouse CD45 (ABclonal, Cat. No. A23709, 1 µg/test), APC/Cyanine7 anti-mouse CD11b (Biolegend, Cat. No. 101226, 1 µg/test), Alexa Fluor™ 488 anti-mouse F4/80 (Invitrogen, Cat. No. 53-4801-80, 1 µg/test), PE anti-mouse CD86 (Biolegend, Cat. No. 105008, 1 µg/test), and APC anti-mouse CD206 (Biolegend, Cat. No. 141708;1 µg/test) were used according to the manufacturer’s instructions.

### High-throughput RNA sequencing

Mouse bone marrow and BMDM cell samples were pooled and the total RNA was isolated to construct the RNA-seq libraries. The RNA libraries were sequenced using an Illumina Hiseq platform (Illumina, San Diego, CA, USA) by Beijing Tsingke Biotech Co., Ltd (Beijing, China). Fragments Per Kilobase of transcript per Million mapped reads (FPKM) were calculated for additional statistics. All reads were mapped to the mouse genome (GRCm38/mm10). The DAVID tool was used for pathway enrichment and gene cluster analysis (https://david.ncifcrf.gov/).

### The glycogen detection

The level of glycogen was measured by Periodic Acid-Schiff (PAS) staining and TEM analysis. For PAS staining, BMDMs were subjected to PAS staining, which included the use of PAS Kit (Beyotime, Nanjing, Jiangsu, China). The BMDMs were photographed with a Nikon microscope (ECLIPSE, Ts2R-FL, Tokyo, Japan). The TEM analysis was performed by Servicebio company (Wuhan, Hubei, China) according to a previous study [30]. Images were taken by using Hitachi HT7800 electron microscope (HT-7800, Hitachi, Tokyo, Japan).

### Statistical analysis

Statistical analysis was conducted using GraphPad (version 9.5, San Diego, CA, USA). The data were presented as mean ± SD (standard deviation) for each group. One-way ANOVA followed by followed by Bonferroni’s *post hoc* test were performed to analyze the data. A *P*-value < 0.05 was considered statistically significant.

## Results

### Systemic Angptl8 is increased in response to CSS signals

**Fig. 1:**
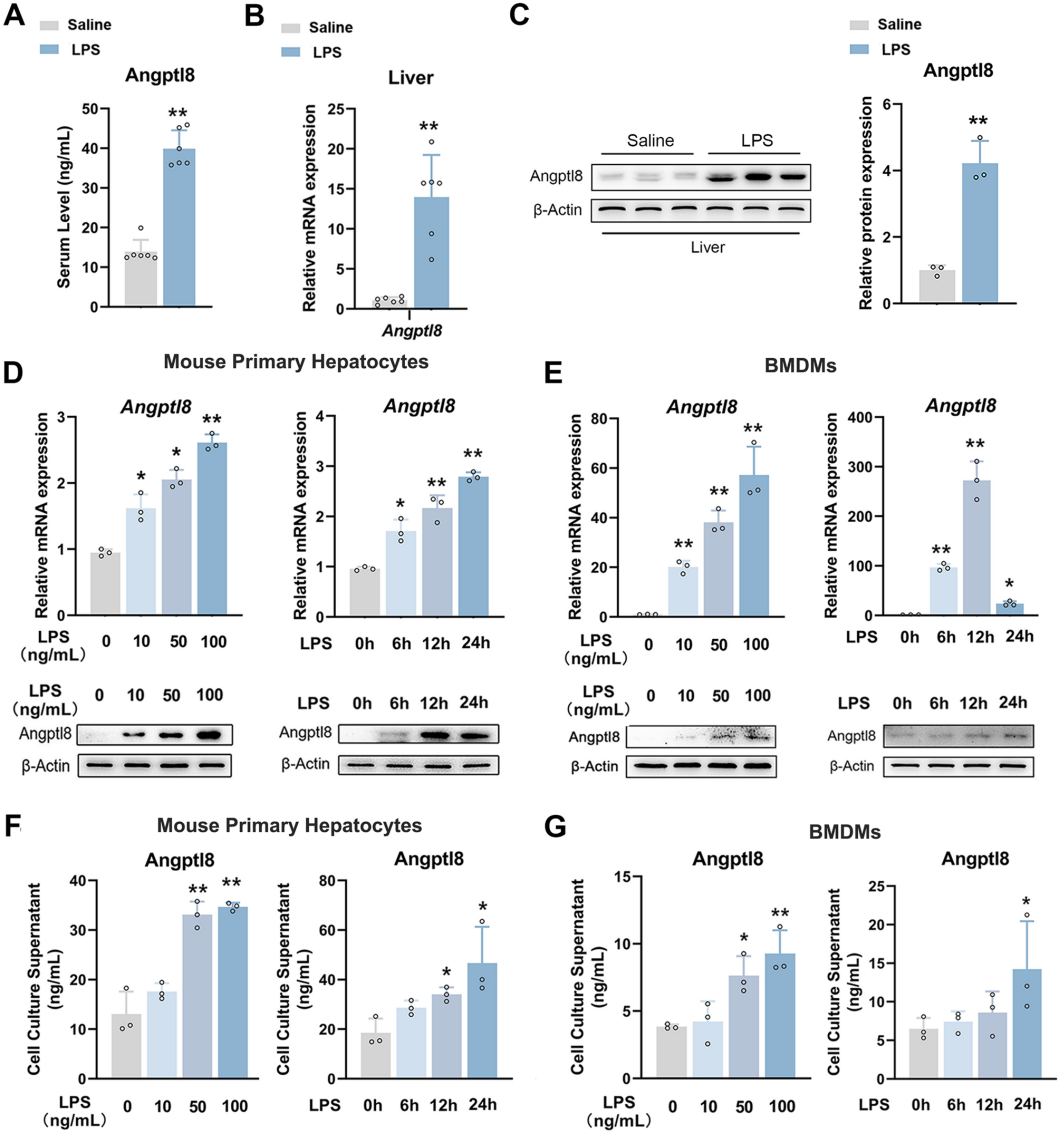
Systemic Angptl8 is increased in response to CSS signals. Mice were subjected with 25 mg/kg LPS for 6 h. **A** Serum Angptl8 levels. **B** The relative mRNA of Angptl8 in mouse liver. ^**^P < 0.01 *v.s*. Saline group, one-way ANOVA followed by Bonferroni’s *posthoc* test, n = 6. **C** The protein expression of Angptl8 in mouse liver. ^**^P < 0.01 *v.s*. Saline group, one-way ANOVA followed by Bonferroni’s *posthoc* test, n = 3. (D) The relative mRNA and protein expression of Angptl8 in mouse PHs treated with indicated doses and time-points of LPS. **E** The relative mRNA and protein expression of Angptl8 in mouse BMDMs treated with indicated doses and time-points of LPS. **F** Supernatant Angptl8 levels in mouse PHs treated with indicated dose and time-points of LPS. **G** Supernatant Angptl8 levels in mouse BMDMs treated with indicated dose and time of LPS. ^*^P < 0.05 and ^**^P < 0.01 *v.s*. No stimulus group, one-way ANOVA followed by Bonferroni’s *posthoc* test, n = 3. All values are presented as the mean ± SD

LPS serves as a potent inflammatory signal capable of inducing CSS. As anticipated, ELISA analysis disclosed a significant increase in serum Angptl8 levels in response to LPS stimulation (Fig. 1A). Given that murine Angptl8 is predominantly secreted by the liver, we subsequently investigated its expression levels in the mouse liver. As shown in Fig. 1B, C, compared to those in the CTL group, the mRNA and protein expression levels of Angptl8 in the livers of LPS-treated mice were significantly elevated by 12.1-fold and 4.2-fold, respectively. To further explore the causal relationships between Angptl8 and CSS in vitro, we stimulated mouse PHs and BMDMs with LPS. RT-qPCR and western blot analyses demonstrated that LPS triggered the mRNA and protein expression of Angptl8 in a dose- and time-dependent manner in both cell types (Figs. 1D, E and S1A, B). Correspondingly, the secreted Angptl8 levels were increased in the supernatants of both mouse primary hepatocytes (PHs) and BMDMs treated with LPS (Fig. 1F, G). In addition, LPS stimulation induced a 14.4-fold increase in the mRNA expression levels of Angptl8 in the bone marrow of mice (Fig. S1C). Collectively, these data imply that Angptl8 may be potentially implicated in the progression of CSS. Mouse primary hepatocytes and BMDMs are the major sources of secreted Angptl8 in the circulatory system of LPS-treated mice.

### Angptl8 deficiency improves survival and inhibits CSS progression in LPS-induced endotoxemic mice

**Fig. 2:**
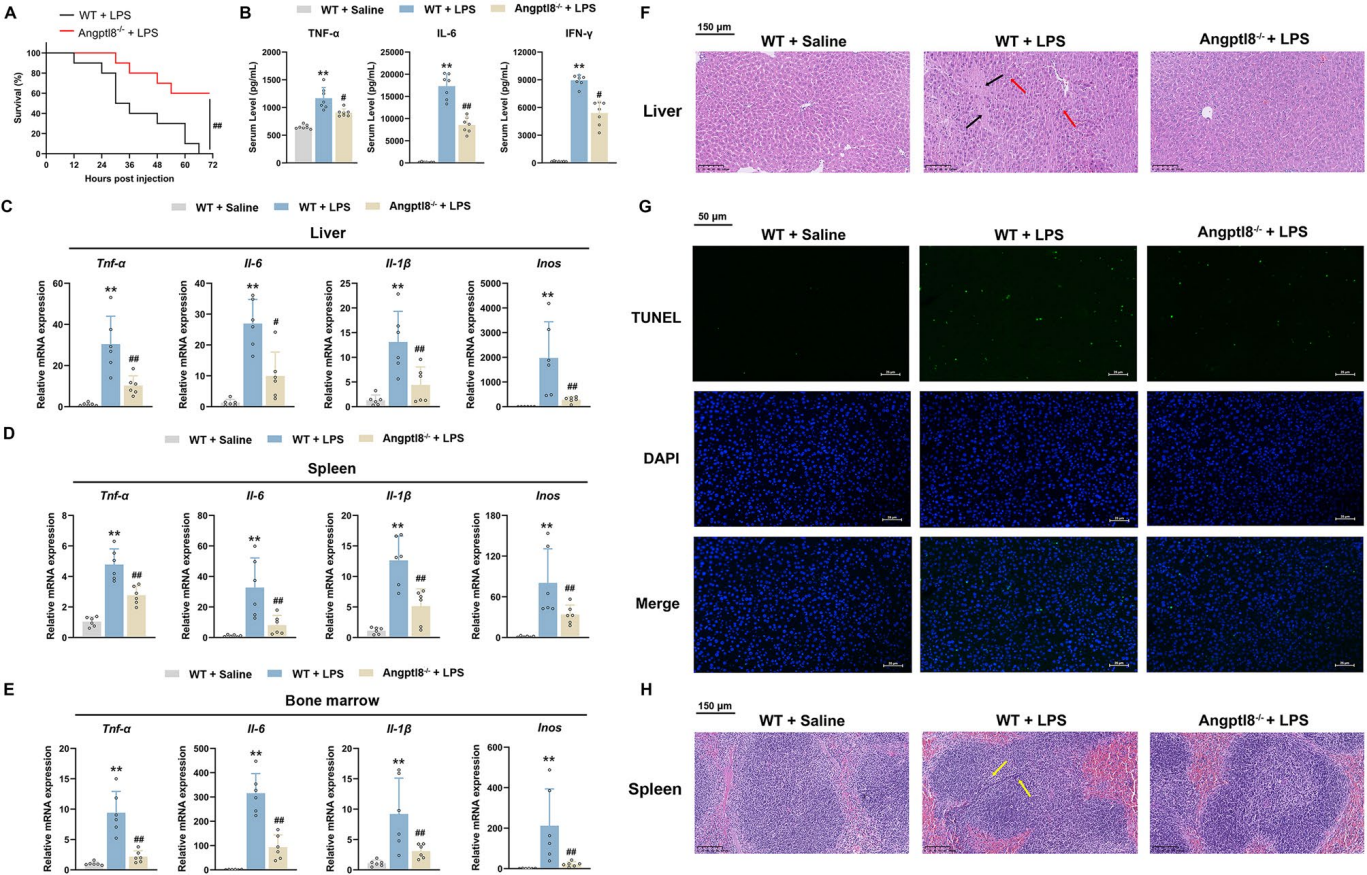
Angptl8 deficiency improves survival and inhibits CSS progression in LPS-induced endotoxemic mice. **A** The survival of the WT and Angptl8^−/−^ mice stimulated with LPS (25 mg/kg) (*i.p.*). ^##^*P* < 0.01 *v.s*. WT + LPS group, one-way ANOVA followed by Bonferroni’s *posthoc* test, n = 10. **B** Measurement of TNF-α, IL-6, IFN-γ secretion in mouse serum by ELISA after treatment with LPS for 6 h. **C**–**E** The relative mRNA expression of *Tnf-α*, *Il-6*, *Il-1β* and *Inos* in mouse **C** liver, **D** spleen and **E** bone marrow. ^**^P < 0.01 *v.s*. WT + Saline group, ^#^*P* < 0.05 and ^##^*P* < 0.01 *v.s*. WT + LPS group, one-way ANOVA followed by Bonferroni’s *posthoc* test, n = 6. **F** Representative images of liver by H&E staining. The black arrows point to hepatocyte necrosis, and the red arrows point to inflammatory cells infiltration. **G** TUNEL assay for liver. **H** Representative images of spleen by H&E staining. The yellow arrows point to fragmentation of spleen cells. All values are presented as the mean ± SD

To further clarify the role of Angptl8 in the progression of LPS-induced acute CSS, we administered a dose of 25 mg/kg LPS to both Angptl8^−/−^ and wild type (WT) mice. As shown in Fig. 2A, such a high dose of LPS led to the death of WT mice within 72 h, whereas genetic ablation of Angptl8 significantly enhanced the survival rate of LPS-treated mice to 60%. On the other hand, the progression of LPS-induced CSS is accompanied by systemic and local inflammation. Consistently, LPS indeed elevated the serum levels of pro-inflammatory cytokines, including TNF-α, IL-6 and IFN-γ. In contrast, Angptl8 deficiency retarded the LPS-induced increase in the levels of these cytokines in the serum of mice (Fig. 2B). Moreover, compared with WT mice, the LPS-induced mRNA expression of pro-inflammatory cytokines, such as *Tnf-α*, *Il-6*, *Il-1β* and *Inos*, was remarkably decreased in the liver, spleen, and bone marrow of Angptl8^−/−^ mice (Fig. 2C–E). Histologically, H&E staining demonstrated that Angptl8 deficiency alleviated the LPS-induced damage to the hepatic lobule structure. This was evidenced by the reduction in bridging necrosis and the infiltration of inflammatory cells in the liver of mice (Fig. 2F). The beneficial effects of Angptl8 deficiency on LPS-induced liver damage were further verified by the TUNEL staining assay. The results revealed that the number of LPS-increased apoptotic cells in the liver of Angptl8^−/−^ mice was markedly decreased compared with that in WT mice (Fig. 2G). In addition, serum levels of LPS-induced elevated ALT and AST were correspondingly reduced in Angptl8^−/−^ mice (Fig. S2). Meanwhile, compared with WT mice, the LPS-triggered pathological changes in the mouse spleen, such as blurred boundaries between the white and red pulps and lymphocyte necrosis, were significantly alleviated in the Angptl8^−/−^ mice (Fig. 2H).

### Angptl8 activates macrophage M1 polarization

**Fig. 3:**
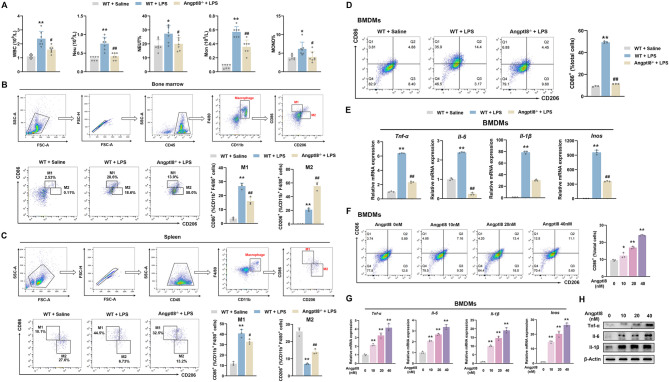
Angptl8 activates macrophage M1 polarization. **A** White blood cell composition and proportion in mice. ^*^P < 0.05 and ^**^P < 0.01 *v.s*. WT + Saline group, ^#^*P* < 0.05 and ^##^*P* < 0.01 *v.s*. WT + LPS group, one-way ANOVA followed by Bonferroni’s *posthoc* test, n = 6. **B**, **C** Percentages of CD86^+^ and CD206^+^ cells detected by flow cytometry in the **B** bone marrow and **C** spleen. **D** Percentages of CD86^+^ cells detected by flow cytometry in BMDMs isolated from WT and Angptl8^−/−^ mice. **E** The relative mRNA expression of *Tnf-α*, *Il-6*, *Il-1β* and *Inos* in BMDMs isolated from WT and Angptl8^−/−^ mice. ^**^P < 0.01 *v.s*. WT + Saline group, ^#^*P* < 0.05 and ^##^*P* < 0.01 *v.s*. WT + LPS group, one-way ANOVA followed by Bonferroni’s *posthoc* test, n = 3. **F** Percentages of CD86^+^ cells detected by flow cytometry in BMDMs treated with recombinant Angptl8 (0, 10, 20, 40 nM) for 24 h. **G** The relative mRNA and **H** protein expression of *Tnf-α*, *Il-6*, *Il-1β* and *Inos* in BMDMs treated with recombinant Angptl8. ^*^P < 0.05 and ^**^P < 0.01 *v.s*. No stimulus group, one-way ANOVA followed by Bonferroni’s *posthoc* test, n = 3. All values are presented as the mean ± SD

Given that inflammatory cytokines are mainly derived from a variety of immune cells such as macrophages and neutrophils, we conducted a CBC analysis. We found that LPS triggered a significant increase in the numbers of white blood cells, neutrophils, and monocytes in the blood of WT mice. More importantly, such an increase was abolished in the blood of Angptl8^−/−^ mice (Fig. 3A). These results suggest that immune cell populations contribute to the beneficial effects of Angptl8 deficiency in counteracting the inflammation and mortality associated with the progression of CSS. It should be noted that macrophages originate from monocytes and are the main cell population responsible for producing pro-inflammatory cytokines, especially in their M1 phenotype [31]. Flow cytometry sorting analysis revealed that Angptl8 deficiency reduced LPS-induced M1 macrophage polarization by 14.7% and 12.0% in the bone marrow and spleen of mice, respectively. Consistent with these results, Angptl8 deficiency correspondingly promoted M2 polarization by 39.4% and 7.5% in the bone marrow and spleen of LPS—treated mice, respectively (Fig. 3B, C).

To validate the role of Angptl8 in macrophage M1 polarization, we isolated BMDMs from the bone marrow of either WT or Angptl8^−/−^ mice and differentiated them into M1 macrophages by stimulating with LPS. Flow cytometry analysis demonstrated that the number of CD86-positive cells increased dramatically in response to LPS stimulation, yet decreased when BMDM cells lacked Angptl8 (Fig. 3D). Accordingly, the mRNA levels of M1 macrophage biomarkers, such as *Tnf-α*, *Il-6*, *Il-1β* and *Inos*, were simultaneously reduced in LPS-treated Angptl8^−/−^ BMDMs compared with those in LPS-treated WT cells (Fig. 3E). To further elucidate the role of Angptl8 in macrophage polarization, we treated BMDMs with recombinant Angptl8 for 24 h. As shown in Fig. 3F, Angptl8 increased the number of CD86-positive M1 macrophages in a dose-dependent manner. Additionally, the mRNA and protein expression levels of M1 macrophage biomarkers were correspondingly elevated (Figs. 3G, H and S3). Importantly, to clarify the impact of Angptl8 deletion on hepatic macrophage polarization, we isolated liver Kupffer cells from Angptl8^−/−^ and WT mice and performed immunofluorescence analyses to evaluate CD86 (M1 marker) and CD206 (M2 marker) expression. Our results showed that LPS stimulation induced a significant increase in CD86 expression and a concomitant decrease in CD206 expression, while these effects that were reversed in Kupffer cells from Angptl8^−/−^ mice (Fig. S4A). Additionally, treatment with recombinant Angptl8 elicited a significant increase in CD86 expression in Kupffer cells (Fig. S4B), indicating a shift toward M1 polarization. These data suggest that Angptl8 is a sufficient and essential factor for triggering macrophage M1 polarization.

### Angptl8 inhibits macrophage M2 polarization

**Fig. 4:**
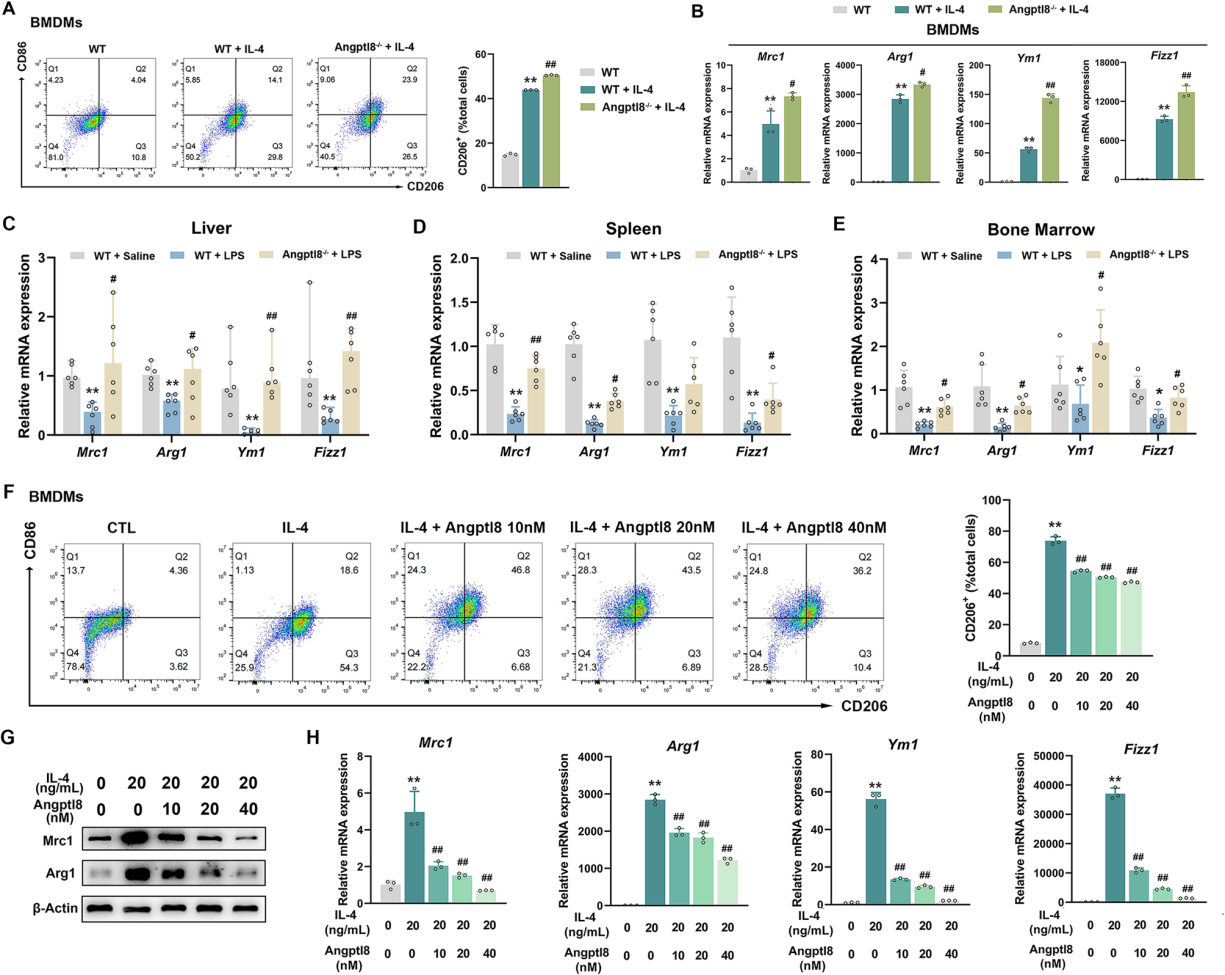
Angptl8 inhibits macrophage M2 polarization in BMDMs. **A** Percentages of CD206^+^ cells detected by flow cytometry in BMDMs isolated from WT and Angptl8^−/−^ mice treated with IL-4. **B** The relative mRNA expression of *Cd206*, *Arg1*, *Ym1* and *Fizz1* in BMDMs. ^**^P < 0.01 *v.s*. No stimulus group, ^#^*P* < 0.05 and ^##^*P* < 0.01 *v.s*. WT + IL-4 group, one-way ANOVA followed by Bonferroni’s *posthoc* test, n = 3. **C**–**E** The relative mRNA expression of *Cd206*, *Arg1*, *Ym1* and *Fizz1* in mouse **C** liver, **D** spleen, **E** bone marrow. ^*^P < 0.05 and ^**^P < 0.01 *v.s*. WT + Saline group, ^#^*P* < 0.05 and ^##^*P* < 0.01 *v.s*. WT + LPS group, one-way ANOVA followed by Bonferroni’s *posthoc* test, n = 6. **F** Percentages of CD206^+^ cells detected by flow cytometry in BMDMs treated with IL-4 and recombinant Angptl8. **G** The relative protein expression of Cd206 and Arg1 in BMDMs detected by Western Blot. **H** The relative mRNA expression of *Cd206*, *Arg1*, *Ym1* and *Fizz1* in BMDMs treated with IL-4 (20 nM) and recombinant Angptl8 (0, 10, 20, 40 nM). ^**^P < 0.01 *v.s*. No stimulus group, ^##^*P* < 0.01 *v.s*. IL-4 group, one-way ANOVA followed by Bonferroni’s *posthoc* test, n = 3. All values are presented as the mean ± SD

Since M2 polarization represents an anti-inflammatory phenotype of macrophages in countering CSS, we differentiated BMDMs into M2 macrophages by stimulating them with IL-4. As expected, IL-4 promoted M2 macrophage polarization, as manifested by an increased percentage of CD206-positive cells, along with the induction of the mRNA and protein expression of Cd206, Arg1, Ym1, and Fizz1, which are characteristic genes of M2 macrophages. Surprisingly, the percentage of CD206-positive cells was further elevated in IL-4-treated BMDMs isolated from Angptl8^−/−^ mice compared to those from WT mice (Fig. 4A). Meanwhile, the expression levels of these characteristic genes were also upregulated in these Angptl8^−/−^ BMDMs (Fig. 4B). Similar results were observed in the liver, spleen and bone marrow of Angptl8^−/−^ mice (Fig. 4C–E). These results indicated that Angptl8 is a potential inhibitor of macrophage M2 polarization. To substantiate this hypothesis, we treated IL-4-treated BMDMs with recombinant Angptl8 for 24 h. Angptl8 inhibited the IL-4-induced macrophage M2 polarization in a concentration-dependent manner. This was evidenced by a reduced percentage of CD206-positive cells and decreased expression levels of the characteristic genes for macrophage M2 polarization (Figs. 4F–H and S5).

### Angptl8 modulates macrophage polarization in BMDMs through regulation of glycogen metabolism

**Fig. 5:**
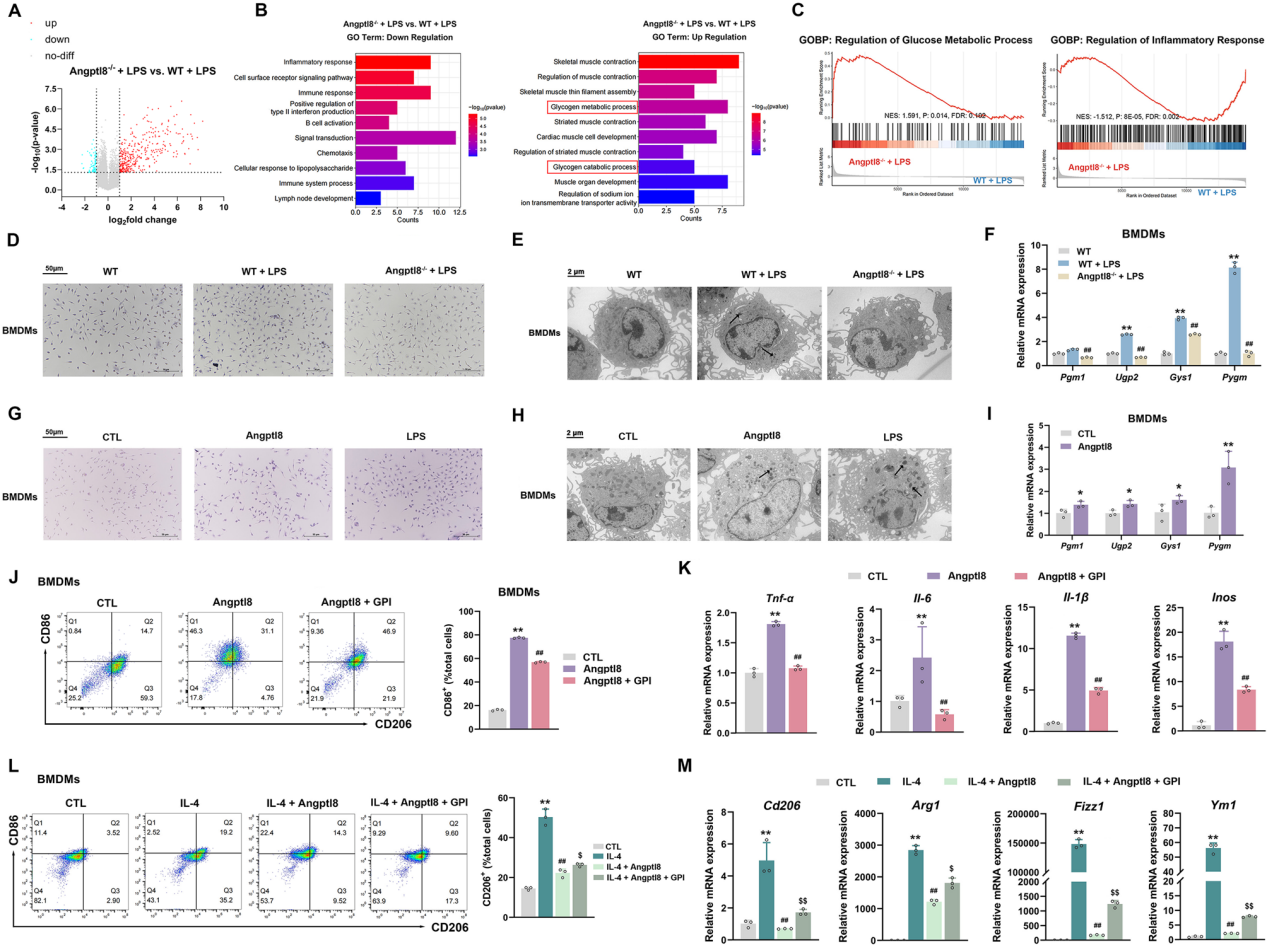
Angptl8 modulates macrophage polarization in BMDMs through regulation of glycogen metabolism. **A** Volcano plot showing differentially expressed genes (DEG) between Angptl8^−/−^+LPS and WT + LPS group. The up-regulated genes (red) with log2_fold change > 1, and the down-regulated genes (blue) with log2_fold change < − 1, and with *P* < 0.05. **B** GO enrichment analysis of down and up regulations for key targets. **C** GSEA of key pathways. **D** Intracellular glycogen levels in BMDMs isolated from WT and Angptl8^−/−^ mice treated with LPS (100 ng/mL) were detected and observed by PAS staining and **E** TEM (The arrows point to intracellular glycogen deposits). **F** The relative mRNA expression of *Pgm1*, *Ugp2* and *Gys1* (glycogen biosynthesis) and *Pygm* (glycogenolysis) in BMDMs isolated from WT and Angptl8^−/−^ mice. ^**^P < 0.01 *v.s*. No stimulus group, ^##^*P* < 0.01 *v.s*. WT + LPS group, one-way ANOVA followed by Bonferroni’s *posthoc* test, n = 3. **G** Intracellular glycogen levels in BMDMs treated with LPS (100 ng/mL) and recombinant Angptl8 (40 nM) were detected and observed by PAS staining and **H** TEM (The arrows point to intracellular glycogen deposits). **I** The relative mRNA expression of *Pgm1*, *Ugp2* and *Gys1* and *Pygm* in BMDMs treated with recombinant Angptl8. ^*^P < 0.05 and ^**^P < 0.01 *v.s*. No stimulus group, one-way ANOVA followed by Bonferroni’s *posthoc* test, n = 3. **J** Percentages of CD86^+^ cells detected by flow cytometry in BMDMs treated with recombinant Angptl8 and GPI (20 µM). **K** The relative mRNA expression of *Tnf-α*, *Il-6*, *Il-1β* and *Inos* in BMDMs. ^**^P < 0.01 *v.s*. No stimulus group, ^##^*P* < 0.01 *v.s*. Angptl8 group, one-way ANOVA followed by Bonferroni’s *posthoc* test, n = 3. **L** Percentages of CD206^+^ cells detected by flow cytometry in BMDMs treated with IL-4, recombinant Angptl8 and GPI. **M** The relative mRNA expression of *Cd206*, *Arg1*, *Ym1* and *Fizz1* in BMDMs. ^**^P < 0.01 *v.s*. No stimulus group, ^##^*P* < 0.01 *v.s*. IL-4 group, ^$^*P* < 0.05 and ^$$^*P* < 0.01 vs. IL-4 + Angptl8 group, one-way ANOVA followed by Bonferroni’s *posthoc* test, n = 3. All values are presented as the mean ± SD

To comprehensively elucidate the downstream molecular events within the transcriptional network orchestrated by Angptl8, transcriptome analysis was performed. The results demonstrated that, in the bone marrow of LPS-treated Angptl8^−/−^ mice, the mRNA expression levels of a total of 438 genes were altered compared with those of LPS-treated WT mice (Fig. 5A). Notably, these genes were predominantly enriched in glycogen metabolism and inflammatory response pathways (Fig. 5B). Additionally, GSEA analysis revealed that Angptl8 deficiency activates glucose metabolism and inhibits inflammatory responses in the bone marrow of LPS-treated mice (Fig. 5C). Moreover, an additional RNA-seq analysis on BMDMs were performed and revealed that a total of 517 genes exhibited differential mRNA expression in BMDMs from LPS-treated Angptl8^−/−^ mice, compared to those from LPS-treated WT mice (Fig. S6A). Notably, these DEGs were primarily enriched immune and inflammatory response pathways (Fig. S6B). Furthermore, GSEA demonstrated that Angptl8 deficiency activates glucose metabolism and suppresses inflammatory responses in BMDMs isolated from LPS-treated mice (Fig. S6C). These data thus further confirm that glycogen metabolism and inflammatory response pathways are key downstream molecular events within the transcriptional network orchestrated by Angptl8 in BMDMs.

Considering that the activation of glycogen metabolism is a characteristic event of the inflammatory metabolic phenotype in macrophages, the relationship between Angptl8 and glycogen content was evaluated. Periodic Acid-Schiff (PAS) staining indicated that LPS induced a significant accumulation of glycogen in BMDMs isolated from WT mice. Conversely, this accumulation was markedly reduced in BMDMs isolated from Angptl8^−/−^ mice (Fig. 5D). Moreover, these results were further validated by TEM analysis (Fig. 5E). At the molecular level, the mRNA expression levels of enzymes involved in glycogen biosynthesis (*Pgm1*, *Ugp2* and *Gys1*) and glycogenolysis (*Pygm*) were correspondingly increased in response to LPS stimulation but decreased in Angptl8-deficient BMDMs (Fig. 5F). These findings suggest that Angptl8 is an essential mediator in LPS-triggered activation of glycogen metabolism. To further clarify the relationship between Angptl8 and activated glycogen metabolism in inflammatory macrophages, the intracellular glycogen levels in BMDMs treated with recombinant Angptl8 for 24 h were examined. Intriguingly, Angptl8 increased the intracellular glycogen levels in BMDMs (Fig. 5G, H). However, only the mRNA expression levels of glycogenolysis enzymes, such as *Pygm*, were increased in response to Angptl8 stimulation, while the mRNA expression levels of enzymes involved in glycogen biosynthesis were only slightly affected (Fig. 5I). Interestingly, no upregulation of the mRNA expression levels of glycogen metabolic genes were observed in hepatocytes treated with recombinant Angptl8 (Fig. S7A). These data imply that over-accumulated Angptl8 is a sufficient factor for the activation of glycogenolysis to initiate the M1-type polarization of macrophages.

On the other hand, to dissect the role of glycogen metabolism, particularly glycogenolysis, in Angptl8-initiated macrophage M1 polarization, GPI was used to inhibit glycogen phosphorylase (GPase) during the glycogenolysis process. It was found that GPI suppressed the Angptl8-triggered macrophage M1 polarization (Figs. 5J, K and S7B) and abolished the inhibitory effect of Angptl8 on IL-4-induced macrophage M2 polarization (Figs. 5L, M and S7C).

### Phosphorylated JNK mediates Angptl8-induced activation of glycogen metabolism and M1 polarization in macrophages

**Fig. 6:**
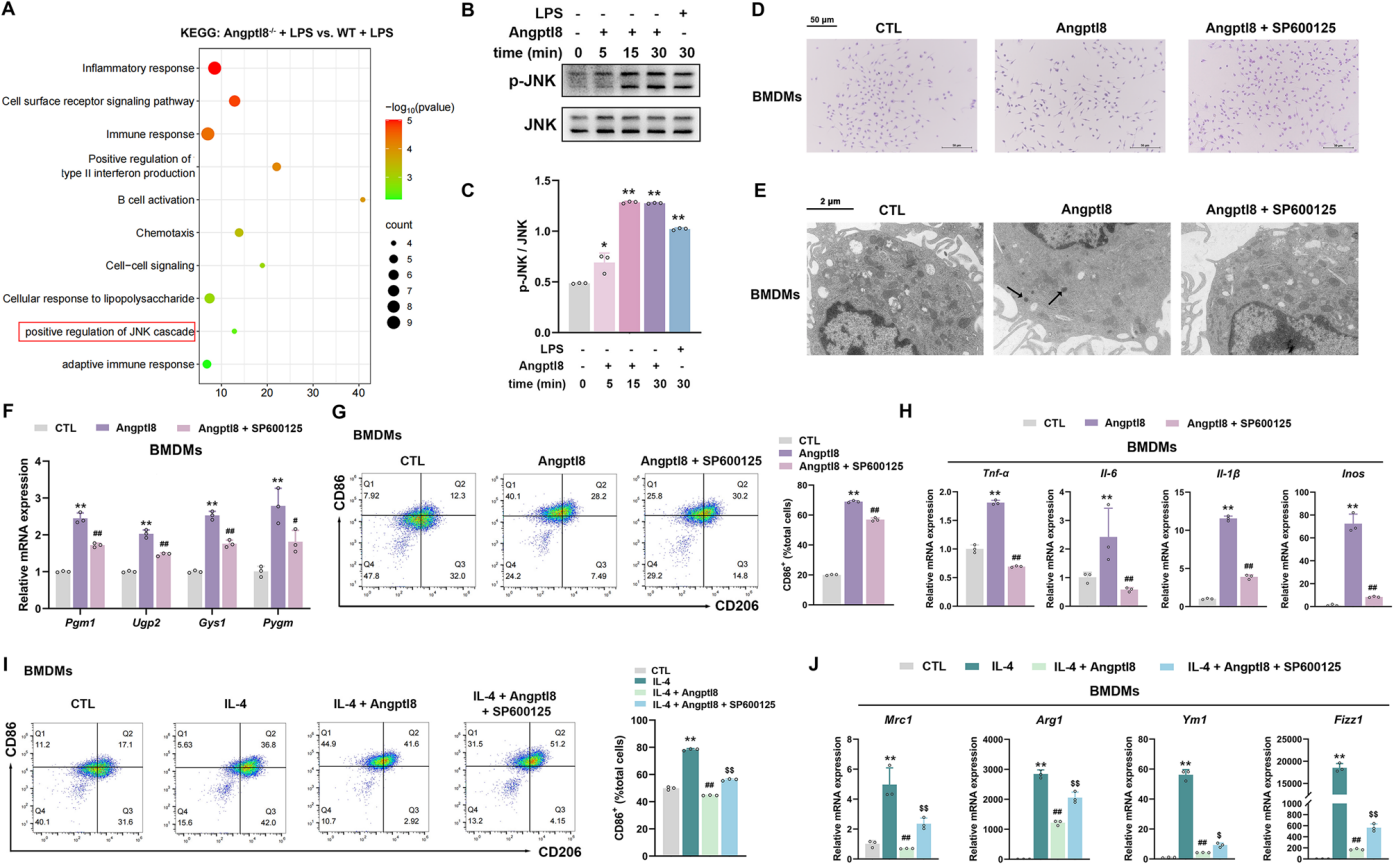
Phosphorylated JNK mediates Angptl8-induced activation of glycogen metabolism and M1 polarization in macrophages. **A** KEGG pathway enrichment analysis of key targets. **B** The protein expression of JNK phosphorylation (p-JNK) and JNK in BMDM treated with recombinant Angptl8 (40 nM) for 0, 5, 15, 30 min, detected by western blot. **C** The quantitative analysis of Relative JNK phosphorylation analyzed by Image J. ^*^P < 0.05 and ^**^P < 0.01 *v.s*. No stimulus group, one-way ANOVA followed by Bonferroni’s *posthoc* test, n = 3. **D** Intracellular glycogen levels in BMDMs treated with recombinant Angptl8 and SP600125 (a JNK inhibitor 10 µM) were detected and observed by PAS staining and **E** TEM (The arrows point to intracellular glycogen deposits). **F** The relative mRNA expression of *Pgm1*, *Ugp2*,* Gys1* and *Pygm* in BMDMs. **G** Percentages of CD86^+^ cells detected by flow cytometry. **H** The relative mRNA expression of *Tnf-α*, *Il-6*, *Il-1β* and *Inos* in BMDMs. ^**^P < 0.01 *v.s*. No stimulus group, ^#^*P* < 0.05 and ^##^*P* < 0.01 *v.s*. Angptl8 group, one-way ANOVA followed by Bonferroni’s *posthoc* test, n = 3. **I** Percentages of CD206^+^ cells in BMDMs treated with IL-4, recombinant Angptl8 and SP600125, detected by flow cytometry. **J** The relative mRNA expression of *Cd206*, *Arg1*, *Ym1* and *Fizz1* in BMDMs. ^**^P < 0.01 *v.s*. No stimulus group, ^##^*P* < 0.01 *v.s*. IL-4 group, ^$^*P* < 0.05 and ^$$^*P* < 0.01 vs. IL-4 + Angptl8 group, one-way ANOVA followed by Bonferroni’s *posthoc* test, n = 3. All values are presented as the mean ± SD

To comprehensively elucidate the molecular mechanism underlying the regulation of glycogen metabolism by Angptl8, a KEGG analysis was meticulously conducted to identify the potential downstream signaling pathways responsive to Angptl8 in the bone marrow of mice. The results revealed a significant enrichment of the JNK signaling pathway (Fig. 6A). Subsequently, an independent Western blot analysis was performed to validate these findings. As shown in Fig. 6B, recombinant Angptl8 was found to augment the phosphorylation levels of JNK. The quantitative data for the phosphorylation of the JNK protein are presented in Fig. 6C. Of particular significance, SP600125, a highly specific JNK inhibitor, effectively abrogated the Angptl8-activated glycogen metabolism. This was evident from the reduced glycogen storage in BMDMs and the decreased mRNA expression levels of glycogenolysis-related genes (Fig. 6D–F). In line with these results, the blockade of JNK phosphorylation significantly attenuated the Angptl8-induced M1 polarization of macrophages. This was demonstrated by a decrease in the proportion of CD86-positive cell populations and reduced mRNA expression levels of pro-inflammatory genes (Fig. 6G ,H). These cellular and molecular changes provided clear evidence for the role of JNK in the macrophage polarization process. Moreover, the inhibitory effects of Angptl8 in antagonizing IL-4-triggered M2 polarization of macrophages were completely abolished by the JNK inhibitor (Fig. 6I, J). To further strengthen the mechanistic narrative, we validated the mRNA expression levels of downstream effectors, such as HIF-1α and PPARα, as well as the intracellular content of metabolic intermediate lactate. We found that treatment with recombinant Angptl8 alone or in combination with the JNK inhibitor SP600125 had a modest effect on *Hif-1α* mRNA expression. In contrast, recombinant Angptl8 significantly inhibited *Pparα* mRNA expression, and this inhibitory effect was blocked by SP600125 (Fig. S8A). Consistent with these results, Angptl8 induced a marked increase in lactate production, while SP600125 partially reduced Angptl8-induced lactate production (Fig. S8B). This finding further emphasized the crucial role of JNK in mediating the complex interplay between Angptl8 and macrophage polarization. Collectively, these data suggest that the JNK MAPK serves as a key mediator of the actions of Angptl8 on glycogen metabolism and the M1 polarization of macrophages.

### Angptl8 neutralization alleviated LPS-triggered CSS

**Fig. 7:**
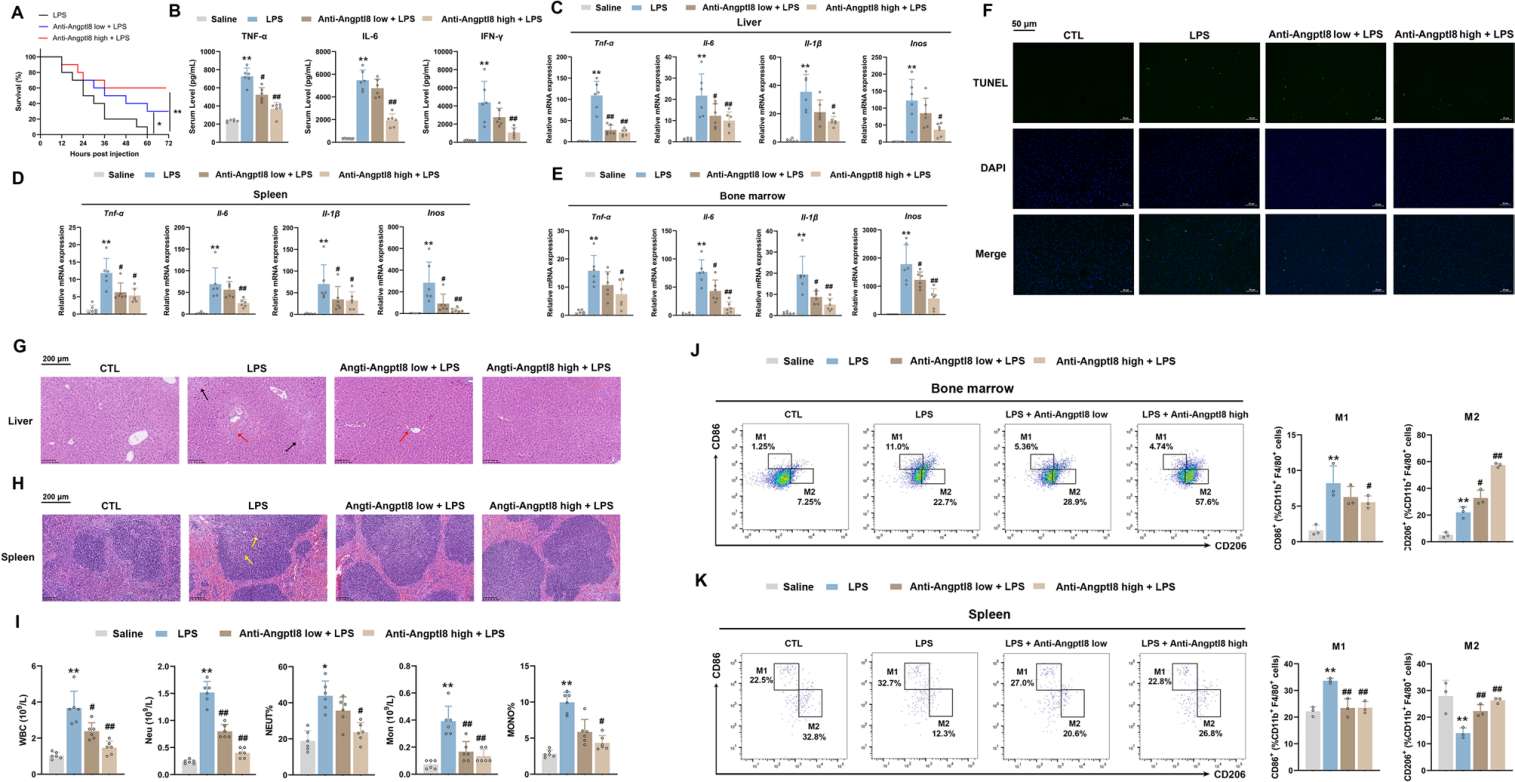
Angptl8 neutralization alleviated LPS-triggered CSS. **A** The survival of mice treated with LPS (25 mg/kg) (*i.p.*) and neutralized Angptl8 antibody (low: 10 µg/25 g; high: 50 µg/25 g) (*i.v.*). ^*^P < 0.05 and ^**^P < 0.01 *v.s*. LPS group, one-way ANOVA followed by Bonferroni’s *posthoc* test, n = 10. **B** Measurement of TNF-α, IL-6, IFN-γ secretion in mouse serum by ELISA. **C**–**E** The relative mRNA expression of *Tnf-α*, *Il-6*, *Il-1β* and *Inos* in mouse **C** liver, **D** spleen and **E** bone marrow. ^**^P < 0.01 *v.s*. Saline group, ^#^*P* < 0.05 and ^##^*P* < 0.01 *v.s*. LPS group, one-way ANOVA followed by Bonferroni’s *posthoc* test, n = 6. **F** Representative images of liver by TUNEL. **G** Representative images of liver by H&E staining (The black arrows point to hepatocyte necrosis, and the red arrows point to inflammatory cells infiltration.) and **H** spleen (The yellow arrows point to fragmentation of spleen cells). **I** White blood cell composition and proportion in the serum of mice. n = 6. **J** Percentages of CD86^+^ and CD206^+^ cells detected by flow cytometry in the bone marrow and **K** spleen. ^**^P < 0.01 *v.s*. Saline group, ^#^*P* < 0.05 and ^##^*P* < 0.01 *v.s*. LPS group, one-way ANOVA followed by Bonferroni’s *posthoc* test, n = 3. All values are presented as the mean ± SD

In light of the in vivo circulating nature of Angptl8, with a translational objective in mind, we implemented a strategy to neutralize the secreted Angptl8 protein. This was achieved by constructing and injecting a neutralizing Angptl8 antibody into the CSS mouse model. As shown in Fig. S9A, the specificity of the neutralizing anti-Angptl8 antibody has been validated via Western blot assay using the mouse liver and multiple liver cell lines. The neutralization of Angptl8 yielded a remarkable outcome, successfully elevating the survival rate of LPS-treated mice to 60% (Fig. 7A). Furthermore, such neutralization effectively abrogated the impact of LPS on the induction of pro-inflammatory cytokines in the mouse serum. Specifically, levels of Tnf-α, Il-6 and IFN-γ were significantly reduced (Fig. 7B). Concurrently, the LPS-activated mRNA expression of pro-inflammatory cytokines, including *Tnf-α*, *Il-6*, *Il-1β* and *Inos*, was markedly decreased in the liver, spleen, and bone marrow of mice treated with the anti-Angptl8 antibody (Fig. 7C–E). The advantageous effects of anti-Angptl8 on LPS-induced liver damage were additionally validated by the TUNEL staining assay (Fig. 7F). Histological analyses provided further evidence for the beneficial effects of anti-Angptl8 in counteracting the progression of LPS-induced CSS in both the liver and spleen (Fig. 7G, H).

CBC analysis demonstrated that the neutralization of Angptl8 effectively abolished the significant increase in the numbers of white blood cells, neutrophils, and monocytes in the blood of mice triggered by LPS (Fig. 7I). More importantly, flow cytometry sorting analysis revealed that anti-Angptl8 led to a reduction in LPS-induced M1 polarization of macrophages by 6.3% and 9.9% in the bone marrow and spleen of mice, respectively (Fig. 7J, K). In accordance with these results, anti-Angptl8 correspondingly induced M2 polarization by 34.9% and 14.5% in the bone marrow and spleen of LPS-treated mice, respectively (Fig. 7J, K).

Finally, to explore the potential toxic effects of anti-Angptl8 on key drug-metabolizing organs, we meticulously examined serological hepatic injury parameters (ALT and AST), renal injury markers (serum creatinine and BUN), and heart injury parameters (ANP and BNP). As shown in Fig. S9B, the neutralization of Angptl8 exerted protective effects on these organs. This was evidenced by the reduced levels of these parameters when mice were pretreated with anti-Angptl8 prior to LPS exposure. Additionally, histological improvement was observed in the kidney, heart, and lung of mice in the anti-Angptl8-treated group (Fig. S9C). Collectively, these findings suggest that the Angptl8-neutralizing antibody holds great promise as a potential candidate for the treatment of CSS progression.

## Discussion

An imbalance in the ratio of M1- and M2-type macrophages constitutes a primary cause of CSS. Furthermore, the liver plays a crucial role in the progression of CSS by transitioning from its metabolic function to an immune function. Consequently, acute phase proteins, which are produced and secreted by the liver, may undergo a comparable transformation, transitioning from metabolic roles to those associated with immune responsiveness and regulation [13–16]. As a hepatokine, Angptl8 exhibits high expression levels in both human and mouse livers and is secreted into the circulatory system following feeding [32]. Furthermore, we have previously demonstrated that short-term refeeding (10 min) potently enhances hepatic protein expression levels, as well as the serum concentration of Angptl8, indicating that Angptl8 is a rapid and responsive hepatokine [21]. Consistent with this observation, we have discovered that LPS induces significant expression of Angptl8 in the liver and bone marrow, and particularly in BMDMs, where LPS triggers a more than 260-fold increase in Angptl8 mRNA expression levels. Notably, we have observed that LPS stimulates the secretion of Angptl8 in the supernatant of BMDMs, suggesting that Angptl8 cannot be solely defined as a hepatokine under inflammatory conditions. On the other hand, under CSS conditions, the majority of macrophages present in the liver are derived from the bone marrow [33]. Consequently, the polarization towards the M1-type of these macrophages may initiate within the bone marrow, where they are stimulated by endogenous Angptl8. Nevertheless, upon migrating into the liver, these activated M1-type macrophages encounter relatively higher concentrations of intracellular Angptl8. This elevated exposure exacerbates the production of pro-inflammatory cytokines, ultimately resulting in organ damage under CSS conditions. Moreover, macrophages account for only ~ 1%-5% of mouse bone marrow cells [34]. Although we observed that LPS significantly induces Angptl8 mRNA expression in bone marrow, whether Angptl8 is expressed in other bone marrow cell types and their responsiveness to LPS warrant further investigation.

Over the past decades, the metabolic regulatory functions of Angptl8 have been gradually illustrated. Specifically, Angptl8 has been found to promote the cleavage of Angptl3, either directly or indirectly, thereby exerting an influence on the activity of lipoprotein lipase LPL and regulating lipid metabolism [20]. In addition, refeeding-induced hepatic Angptl8 serves as an innovative *Zeitegber*, resetting the hepatic circadian clock and modulating lipid metabolism [21]. These findings highlight the pivotal role of Angptl8 in the pathogenesis of metabolic disorders. Given that chronic inflammation is known to aggravate the metabolic disorders, the relationship between chronic inflammation and Angptl8 has been documented. Hepatic Angptl8 and its receptor PirB were increased in NASH patients, while hepatocyte-secreted Angptl8 promotes monocyte-derived macrophage migration and activation by binding to PirB receptor, triggering nuclear translocation of NF-κB p65 subunit and phosphorylation of p38 MAPK and ERK1/2 proteins, resulting in a feedback activation of lipid storage in hepatocytes [24]. Notably, monocyte-derived macrophage recruitment is not only a critical pathogenic event in NASH progression, but also in CSS progression. Consistently, we found that recombinant Angptl8 triggered M1-type macrophage polarization, while suppressed M2-type macrophage polarization. Differently, our findings revealed that the source of Angptl8 for M1-type macrophage polarization is derived from both the hepatocytes and inflammatory macrophages. More importantly, our RNA-seq data indicated only the JNK pathway were enriched in Angptl8-deficient mice. Such an necessitate role of JNK in relaying Angptl8 signals to macrophage activation were further determined by using JNK inhibitor SP600125. These contradictory results may be explained by the different organ immune microenvironment in the liver and bone marrow, as well as the different inflammatory intensity within NASH and CSS progressions.

Given these limitations, we have not yet provided direct experimental evidence that macrophages are the primary source of these cytokines in the liver, spleen, or bone marrow. However, our current work focuses on defining Angptl8’s role in regulating systemic and tissue-specific inflammatory responses, and supporting data indicate that macrophage polarization is a key downstream event. Consistent with established literature, macrophages are widely recognized as major producers of pro-inflammatory cytokines (e.g., TNF-α, IL-6) in these tissues during acute inflammatory stimuli such as LPS-induced CSS [35]. This is particularly relevant in the liver, which contains the largest macrophage pool (~ 80% of total tissue macrophages) comprising resident Kupffer cells and monocyte-derived macrophages; this pool expands rapidly during injury or inflammation, further supporting their potential contribution to cytokine secretion [36, 37]. Collectively, this existing knowledge strongly supports the notion that macrophages drive the cytokine changes observed in our studies. The direct evidence, such as cytokine secretion assays using sorted macrophages from these tissues, would further solidify this link. This important question will be rigorously addressed in our subsequent studies.

Although recent studies have noticed the relationship between Angptl8 and M1-type macrophage polarization, the detailed metabolic status within the macrophages were not addressed. In our study, we clustered active glycogen metabolism within the bone marrow of Angptl8-deficient mice. Indeed, macrophages, by virtue of their mobilizing glycogen metabolism, are polarized to an inflammatory phenotype [38–40]. This metabolic pathway not only enhance the PPP that provides NADPH for antioxidation and cell survival, but also triggers UDPG-P2Y14 signaling pathway to promote the M1-type transition [41]. Under CSS condition, bone marrow-derived Angptl8 firstly initiated the M1-type transition of macrophages, triggering their activation and migration into key organs, such as liver and kidney, where existing abundant glycogen, glucose as well as Angptl8. Hence, with the high metabolic rate of glycogen and sufficient sources (glucose and Angptl8), the macrophages continuously produced pro-inflammatory cytokines, ultimately resulting in organ damage. More importantly, blockage of glycogen metabolism by GPI abrogated the activation effects of Angptl8 on the macrophage M1-type polarization, as well as the detrimental effects of Angptl8 on the macrophage M2 polarization. These results suggest that in addition to its traditional lipid-regulatory function, Angptl8 hold promise to maintain the glycogen homeostasis in the macrophage. In addition, we noticed that Angptl8-JNK axis plays a pivotal role in regulating the expression of PPARα, a key lipid metabolic gene associated with lipid accumulation in macrophages [42]. Considering that Angptl8 is a critical factor involved in triglyceride metabolism [43], further research into the role of Angptl8-driven macrophage M1 polarization from the view of PPARα-orchestrated lipid homeostasis is of particular interest. On the other hand, we did not observe the active glycogen metabolism within the hepatocytes when treated with recombinant Angptl8. This discrepancy is potentially due to the different function of JNK signals in either hepatocytes or macrophages. Briefly, in hepatocytes, activation of JNK signals promotes apoptosis and inhibits insulin resistance, while in macrophages, the presence of JNK promotes obesity-induced insulin resistance and inflammation [44]. In our study, we found that inhibition of JNK protein phosphorylation by SP600125 blocks the Angptl8-activated lactate production, glycogen metabolism and M1-type polarization. Hence, Further studies assessing mitochondrial function or glycolytic flux in macrophages will help clarify how Angptl8-driven glycogenolysis intersects with other metabolic pathways. Collectively, these data suggest Angptl8 possesses a cell-specific mechanism to regulate glycogen metabolism in a manner dependent on the phosphorylation of JNK protein.

Notably, it is reported that intracellular Angptl8 deficiency enhances NF-κB activation and exacerbates inflammatory responses, with Angptl8 self-oligomerization being essential for this regulatory effect. Notably, these inhibitory actions of intracellular Angptl8 were observed in cancer cells (e.g., HepG2), where Angptl8 is highly expressed intracellularly and its self-oligomerization is robustly activated [22, 45]. Thus, we hypothesize that the apparent discrepancy may stem from functional divergence between intracellular and secreted Angptl8. Such localization-dependent functional specialization of a protein in its intracellular vs. secreted forms is well-documented. For example, ISG15, an interferon-induced modifier, exists in both intracellular and extracellular pools: secreted ISG15 exerts cytokine-like activities [46], whereas intracellular ISG15 mediates protein conjugation via ISGylation, which restrains IFN-α/β-dependent autoinflammation [47]. Consistent with this paradigm, our own work supports distinct roles for Angptl8 based on its localization. Specifically, we previously demonstrated that in mouse hepatocytes, secreted Angptl8 reduces the tyrosine phosphorylation of its receptor PirB, thereby diminishing the recruitment of SH2-containing phosphatases (e.g., SHP-1/2) and ultimately activating NF-κB [21]. This aligns with the finding that secreted Angptl8 activates NF-κB in monocyte-derived macrophages during NASH progression [25]. Furthermore, in the current study, we did not focus on the NF-κB signaling pathway, since it was not enriched by either GO or KEGG analysis. Instead, we found that Angptl8-triggered the activation of JNK signaling, which constitutes the core signaling pathway that mediates the inflammatory phenotype of macrophages [48, 49], while JNK inhibitor SP600125 alleviates Angptl8-induced macrophage M1 polarization. Hence, these cumulative observations collectively support our conclusion that Angptl8 deficiency alleviates LPS-induced CSS.

Our findings confirmed an indispensable role of Angptl8 in the M1-type activation of macrophages, while Angptl8 deficiency remarkably alleviated LPS-induced CSS progression in mice. Targeting Angptl8 could be a new strategy against the CSS, at least prolonging the therapeutic window. As a secreted protein from both hepatocytes and macrophages under CSS condition, a neutralized antibody targeting Angptl8 is more favor to synthesized. Previously, we have applied this Anti-Angptl8 to antagonize the food-driven resetting of hepatic circadian clock in mice [21]. In this study, we further resolved the amino acid sequence of Anti-Angptl8, while comprehensively evaluated its effectiveness in mitigating LPS-induced CSS progression. This neutralizing antibody effectively reduced the high mortality rate caused by LPS in mice, decreased M1 macrophage polarization and increased the proportion of M2 macrophages in the bone marrow and spleen. Additionally, serological analyses and H&E staining assays demonstrated that the Angptl8 neutralizing antibody significantly ameliorated multi-organ damage induced by LPS, encompassing the liver, kidneys, heart, and lungs. Hence, Angptl8 neutralization antibody may serve as a promising candidate for CSS patients suffering from severe illnesses such as those caused by viral infections like COVID-19.

**Fig. 8:**
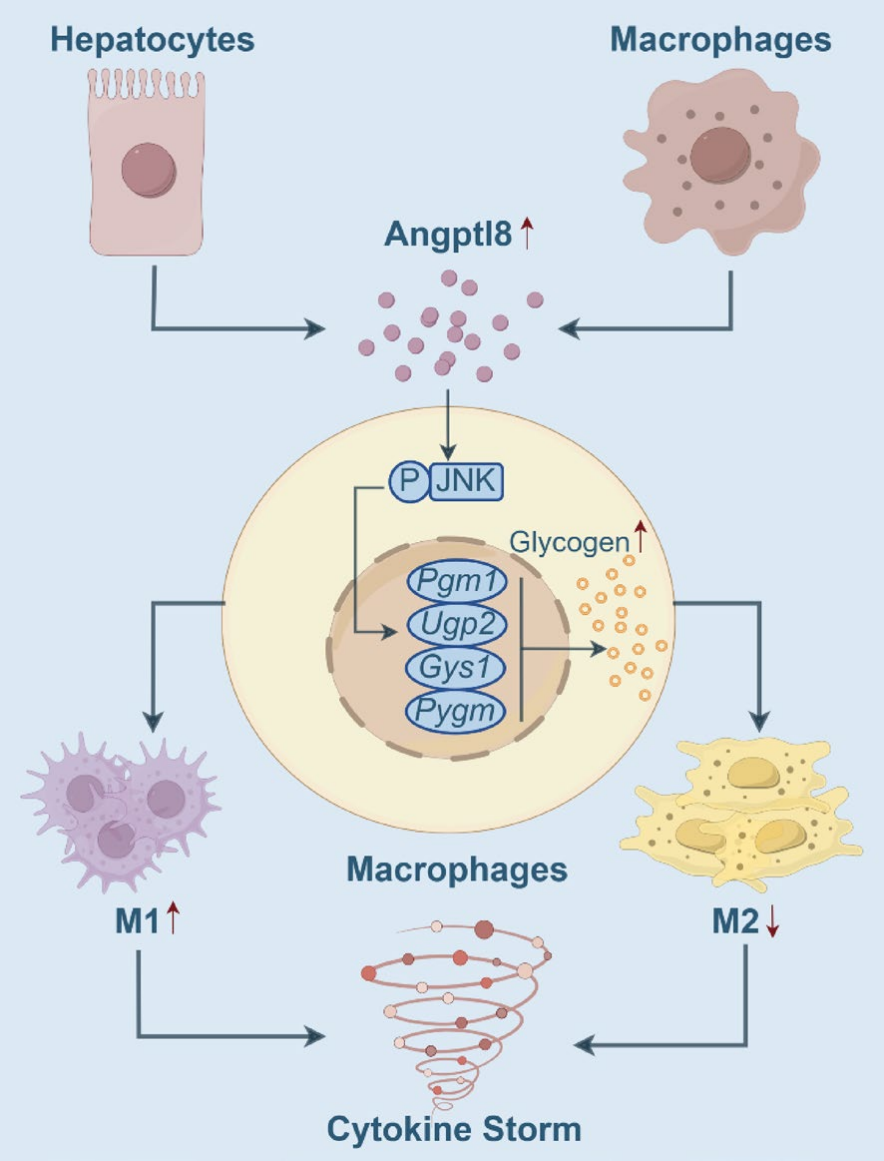
A schematic model illustrating that targeting hepatokine Angptl8 alleviates cytokine storm via macrophage metabolic reprogramming in mice

In summary, we have determined a precise transition of well-established metabolic regulator Angptl8 to its immune regulatory function during the LPS-induced CSS progression (Fig. 8). Our findings illustrate the transformation of a metabolic regulator into an immune regulator from the perspective of Angptl8-driven metabolic reprogramming. Angptl8 emerges as a promising target for the treatment of CSS. Additionally, the Angptl8 neutralizing antibody potentially offers therapeutic advantages for patients, especially those affected by viral infections and immune therapies.

## Supplementary Information

Below is the link to the electronic supplementary material.

Supplementary Material 1

Supplementary Material 2

## Data Availability

The data that support this study are available within the article and its supplementary data files or available from the authors upon request.Uncropped blot images are provided in Supplementary Material 2.

## References

[1] Ramasamy S, Subbian S. Critical determinants of cytokine storm and type I interferon response in COVID-19 pathogenesis. Clin Microbiol Rev. 2021;34(3):10-1128. 10.1128/CMR.00299-20 PMC8142516 33980688

[2] Wang C, Xie J, Zhao L, Fei X, Zhang H, Tan Y, Nie X, Zhou L, Liu Z, Ren Y, et al. Alveolar macrophage dysfunction and cytokine storm in the pathogenesis of two severe COVID-19 patients. EBioMedicine. 2020;57:102833. 32574956 10.1016/j.ebiom.2020.102833 PMC7305897

[3] Sharma C, Ganigara M, Galeotti C, Burns J, Berganza FM, Hayes DA, Singh-Grewal D, Bharath S, Sajjan S, Bayry J. Multisystem inflammatory syndrome in children and Kawasaki disease: a critical comparison. Nat Rev Rheumatol. 2021;17(12):731–48. 34716418 10.1038/s41584-021-00709-9 PMC8554518

[4] Li A, Ling L, Qin H, Arabi YM, Myatra SN, Egi M, Kim JH, Mat Nor MB, Son DN, Fang WF, et al. Epidemiology, Management, and outcomes of sepsis in ICUs among countries of differing national wealth across Asia. Am J Respir Crit Care Med. 2022;206(9):1107–16. 35763381 10.1164/rccm.202112-2743OC

[5] Van Wyngene L, Vanderhaeghen T, Timmermans S, Vandewalle J, Van Looveren K, Souffriau J, Wallaeys C, Eggermont M, Ernst S, Van Hamme E, et al. Hepatic PPARα function and lipid metabolic pathways are dysregulated in polymicrobial sepsis. EMBO Mol Med. 2020;12(2):e11319. 31916705 10.15252/emmm.201911319 PMC7005534

[6] Van Wyngene L, Vandewalle J, Libert C. Reprogramming of basic metabolic pathways in microbial sepsis: therapeutic targets at last? EMBO Mol Med. 2018;10(8):e8712. 10.15252/emmm.201708712 PMC6079534 29976786

[7] Zhang YY, Ning BT. Signaling pathways and intervention therapies in sepsis. Signal Transduct Target Ther. 2021;6(1):407. 34824200 10.1038/s41392-021-00816-9 PMC8613465

[8] Zhu J, Jin Z, Wang J, Wu Z, Xu T, Tong G, Shen E, Fan J, Jiang C, Wang J et al. FGF21 ameliorates septic liver injury by restraining Proinflammatory macrophages activation through the autophagy/HIF-1alpha axis. J Adv Res. 2024;69:477–94. 10.1016/j.jare.2024.04.004 PMC11954821 38599281

[9] Chen LYC, Hoiland RL, Stukas S, Wellington CL, Sekhon MS. Confronting the controversy: interleukin-6 and the COVID-19 cytokine storm syndrome. Eur Respir J. 2020;56(4):2003006. 10.1183/13993003.03006-2020 PMC7474149 32883678

[10] Copaescu A, Smibert O, Gibson A, Phillips EJ, Trubiano JA. The role of IL-6 and other mediators in the cytokine storm associated with SARS-CoV-2 infection. J Allergy Clin Immunol. 2020;146(3):518–34. 32896310 10.1016/j.jaci.2020.07.001 PMC7471766

[11] Yan H, Liu Y, Li X, Yu B, He J, Mao X, Yu J, Huang Z, Luo Y, Luo J et al. Leucine alleviates cytokine storm syndrome by regulating macrophage polarization via the mTORC1/LXRalpha signaling pathway. Elife 2024, 12:RP89750. 10.7554/eLife.89750 PMC10942637 38442142

[12] Schulert GS, Grom AA. Macrophage activation syndrome and cytokine-directed therapies. Best Pract Res Clin Rheumatol. 2014;28(2):277–92. 24974063 10.1016/j.berh.2014.03.002 PMC4074772

[13] Strnad P, Tacke F, Koch A, Trautwein C. Liver—guardian, modifier and target of sepsis. Nat Rev Gastroenterol Hepatol. 2017;14(1):55–66. 27924081 10.1038/nrgastro.2016.168

[14] Choudhury SR, Babes L, Rahn JJ, Ahn BY, Goring KR, King JC, Lau A, Petri B, Hao X, Chojnacki AK, et al. Dipeptidase-1 is an adhesion receptor for neutrophil recruitment in lungs and liver. Cell. 2019;178(5):1205–e12211217. 31442408 10.1016/j.cell.2019.07.017

[15] Hou J, Zhang J, Cui P, Zhou Y, Liu C, Wu X, Ji Y, Wang S, Cheng B, Ye H et al. TREM2 sustains macrophage-hepatocyte metabolic coordination in nonalcoholic fatty liver disease and sepsis. J Clin Invest 2021, 131(4):e135197. 10.1172/JCI135197 PMC7880419 33586673

[16] Choi H, Kim Y, Mirzaaghasi A, Heo J, Kim YN, Shin JH, Kim S, Kim NH, Cho ES, In Yook J et al. Exosome-based delivery of super-repressor IkappaBalpha relieves sepsis-associated organ damage and mortality. *Sci Adv* 2020, 6(15):eaaz6980. 10.1126/sciadv.aaz6980 PMC7141819 32285005

[17] Wang Y, Quagliarini F, Gusarova V, Gromada J, Valenzuela DM, Cohen JC, Hobbs HH. Mice lacking ANGPTL8 (Betatrophin) manifest disrupted triglyceride metabolism without impaired glucose homeostasis. Proc Natl Acad Sci USA. 2013;110(40):16109–14. 24043787 10.1073/pnas.1315292110 PMC3791734

[18] Zhang R. Lipasin, a novel nutritionally-regulated liver-enriched factor that regulates serum triglyceride levels. Biochem Biophys Res Commun. 2012;424(4):786–92. 22809513 10.1016/j.bbrc.2012.07.038

[19] Ren G, Kim JY, Smas CM. Identification of RIFL, a novel adipocyte-enriched insulin target gene with a role in lipid metabolism. Am J Physiol Endocrinol Metab. 2012;303(3):E334–E351. 22569073 10.1152/ajpendo.00084.2012 PMC3423120

[20] Zhang R, Zhang K. An updated ANGPTL3-4-8 model as a mechanism of triglyceride partitioning between fat and oxidative tissues. Prog Lipid Res. 2022;85:101140. 34793860 10.1016/j.plipres.2021.101140 PMC8760165

[21] Chen S, Feng M, Zhang S, Dong Z, Wang Y, Zhang W, Liu C. Angptl8 mediates food-driven resetting of hepatic circadian clock in mice. Nat Commun. 2019;10(1):3518. 31388006 10.1038/s41467-019-11513-1 PMC6684615

[22] Zhang Y, Guo X, Yan W, Chen Y, Ke M, Cheng C, Zhu X, Xue W, Zhou Q, Zheng L, et al. ANGPTL8 negatively regulates NF-kappaB activation by facilitating selective autophagic degradation of IKKgamma. Nat Commun. 2017;8(1):2164. 29255244 10.1038/s41467-017-02355-w PMC5735157

[23] Feng Y, Luo S, Fang C, Ma S, Fan D, Chen Y, Chen Z, Zheng X, Tang Y, Duan X, et al. ANGPTL8 deficiency attenuates lipopolysaccharide-induced liver injury by improving lipid metabolic dysregulation. J Lipid Res. 2024;65(8):100595. 39019343 10.1016/j.jlr.2024.100595 PMC11364043

[24] Zhang Z, Yuan Y, Hu L, Tang J, Meng Z, Dai L, Gao Y, Ma S, Wang X, Yuan Y, et al. ANGPTL8 accelerates liver fibrosis mediated by HFD-induced inflammatory activity via LILRB2/ERK signaling pathways. J Adv Res. 2023;47:41–56. 36031141 10.1016/j.jare.2022.08.006 PMC10173191

[25] Li DP, Huang L, Kan RR, Meng XY, Wang SY, Zou HJ, Guo YM, Luo PQ, Pan LM, Xiang YX, et al. LILRB2/PirB mediates macrophage recruitment in fibrogenesis of nonalcoholic steatohepatitis. Nat Commun. 2023;14(1):4436. 37481670 10.1038/s41467-023-40183-3 PMC10363120

[26] Kircheis R, Haasbach E, Lueftenegger D, Heyken WT, Ocker M, Planz O. NF-κB pathway as a potential target for treatment of critical stage COVID-19 patients. Front Immunol. 2020;11:598444. 33362782 10.3389/fimmu.2020.598444 PMC7759159

[27] Fu ZY, Abou-Samra AB, Zhang R. A lipasin/Angptl8 monoclonal antibody lowers mouse serum triglycerides involving increased postprandial activity of the cardiac lipoprotein lipase. Sci Rep 2015;5:18502. 10.1038/srep18502 PMC4685196 26687026

[28] Chen S, Qian J, Shi X, Gao T, Liang T, Liu C. Control of hepatic gluconeogenesis by the promyelocytic leukemia zinc finger protein. Mol Endocrinol. 2014;28(12):1987–98. 25333514 10.1210/me.2014-1164 PMC5414782

[29] Aparicio-Vergara M, Tencerova M, Morgantini C, Barreby E, Aouadi M. Isolation of Kupffer cells and hepatocytes from a single mouse liver. Methods Mol Biol. 2017;1639:161–71. 28752456 10.1007/978-1-4939-7163-3_16

[30] Luo H, Jiang M, Lian G, Liu Q, Shi M, Li TY, Song L, Ye J, He Y, Yao L, et al. AIDA selectively mediates downregulation of fat synthesis enzymes by ERAD to retard intestinal fat absorption and prevent obesity. Cell Metab. 2018;27(4):843–e853846. 29617643 10.1016/j.cmet.2018.02.021

[31] Bashir S, Sharma Y, Elahi A, Khan F. Macrophage polarization: the link between inflammation and related diseases. Inflamm Res. 2016;65(1):1–11. 26467935 10.1007/s00011-015-0874-1

[32] Zhang R. The ANGPTL3-4-8 model, a molecular mechanism for triglyceride trafficking. Open Biol. 2016;6(4):150272. 27053679 10.1098/rsob.150272 PMC4852456

[33] Serbina NV, Pamer EG. Monocyte emigration from bone marrow during bacterial infection requires signals mediated by chemokine receptor CCR2. Nat Immunol. 2006;7(3):311–7. 16462739 10.1038/ni1309

[34] Hensel JA, Khattar V, Ashton R, Ponnazhagan S. Characterization of immune cell subtypes in three commonly used mouse strains reveals gender and strain-specific variations. Lab Invest. 2019;99(1):93–106. 30353130 10.1038/s41374-018-0137-1 PMC6524955

[35] Hamidzadeh K, Christensen SM, Dalby E, Chandrasekaran P, Mosser DM. Macrophages and the recovery from acute and chronic inflammation. Annu Rev Physiol. 2017;79:567–92. 27959619 10.1146/annurev-physiol-022516-034348 PMC5912892

[36] Ju C, Tacke F. Hepatic macrophages in homeostasis and liver diseases: from pathogenesis to novel therapeutic strategies. Cell Mol Immunol. 2016;13(3):316–27. 26908374 10.1038/cmi.2015.104 PMC4856798

[37] Tacke F, Zimmermann HW. Macrophage heterogeneity in liver injury and fibrosis. J Hepatol. 2014;60(5):1090–6. 24412603 10.1016/j.jhep.2013.12.025

[38] Mills EL, Kelly B, Logan A, Costa ASH, Varma M, Bryant CE, Tourlomousis P, Dabritz JHM, Gottlieb E, Latorre I, et al. Succinate dehydrogenase supports metabolic repurposing of mitochondria to drive inflammatory macrophages. Cell. 2016;167(2):457–70. e413. 27667687 10.1016/j.cell.2016.08.064 PMC5863951

[39] Tannahill GM, Curtis AM, Adamik J, Palsson-McDermott EM, McGettrick AF, Goel G, Frezza C, Bernard NJ, Kelly B, Foley NH, et al. Succinate is an inflammatory signal that induces IL-1beta through HIF-1alpha. Nature. 2013;496(7444):238–42. 23535595 10.1038/nature11986 PMC4031686

[40] Haschemi A, Kosma P, Gille L, Evans CR, Burant CF, Starkl P, Knapp B, Haas R, Schmid JA, Jandl C, et al. The sedoheptulose kinase CARKL directs macrophage polarization through control of glucose metabolism. Cell Metab. 2012;15(6):813–26. 22682222 10.1016/j.cmet.2012.04.023 PMC3370649

[41] Ma J, Wei K, Liu J, Tang K, Zhang H, Zhu L, Chen J, Li F, Xu P, Chen J, et al. Glycogen metabolism regulates macrophage-mediated acute inflammatory responses. Nat Commun. 2020;11(1):1769. 32286295 10.1038/s41467-020-15636-8 PMC7156451

[42] Ye GZ, Gao H, Lin Y, Ding DX, Liao X, Zhang H, Chi YL, Dong SJ. Peroxisome proliferator-activated receptor A/G reprogrammes metabolism associated with lipid accumulation in macrophages. Metabolomics 2019, 15(3):36. 10.1007/s11306-019-1485-6 30830452

[43] Sylvers-Davie KL, Davies BSJ. Regulation of lipoprotein metabolism by ANGPTL3, ANGPTL4, and ANGPTL8. Am J Physiol-Endoc M. 2021;321(4):E493–508. 10.1152/ajpendo.00195.2021 PMC8560382 34338039

[44] Zeke A, Misheva M, Remenyi A, Bogoyevitch MA. JNK signaling: regulation and functions based on complex protein–protein partnerships. Microbiol Mol Biol Rev. 2016;80(3):793–835. 27466283 10.1128/MMBR.00043-14 PMC4981676

[45] Wang CC, Tong Y, Wen YK, Cai J, Guo H, Huang LF, Xu M, Feng MX, Chen XS, Zhang JJ, et al. Hepatocellular carcinoma-associated protein TD26 interacts and enhances sterol regulatory element-binding protein 1 activity to promote tumor cell proliferation and growth. Hepatology. 2018;68(5):1833–50. 29663480 10.1002/hep.30030

[46] Bogunovic D, Byun M, Durfee LA, Abhyankar A, Sanal O, Mansouri D, Salem S, Radovanovic I, Grant AV, Adimi P, et al. Mycobacterial disease and impaired IFN-γ immunity in humans with inherited ISG15 deficiency. Science. 2012;337(6102):1684–8. 22859821 10.1126/science.1224026 PMC3507439

[47] Zhang XQ, Bogunovic D, Payelle-Brogard B, Francois-Newton V, Speer SD, Yuan C, Volpi S, Li Z, Sanal O, Mansouri D, et al. Human intracellular ISG15 prevents interferon-α/β over-amplification and auto-inflammation. Nature. 2015;517(7532):89–U229. 25307056 10.1038/nature13801 PMC4303590

[48] Rao KMK. MAP kinase activation in macrophages. J Leukoc Biol. 2001;69(1):3–10. 11200064

[49] DeFranco AL, Hambleton J, McMahon M, Weinstein SL. Examination of the role of MAP kinase in the response of macrophages to lipopolysaccharide. Prog Clin Biol Res. 1995;392:407–20. 8524948

