# Supplementary material for: Angiopoietin-like protein 8 orchestrates macrophage glycogen metabolism and polarization via the JNK signaling pathway in cytokine storm syndrome
Source: Cell Biosci. 2025 Oct 15;15:140. doi: 10.1186/s13578-025-01487-7 (PMC12522911; doi:10.1186/s13578-025-01487-7)
Supplement: Supplementary file 1 — Supplementary Material 1 [file 13578_2025_1487_MOESM1_ESM.docx]

**Supplementary Information**

**Angiopoietin-like protein 8 Orchestrates Macrophage Glycogen Metabolism and Polarization via the JNK Signaling Pathway in Cytokine Storm Syndrome**

Yang Su^1,†^, Rongtian Zhang^1,†^, Kongdong Li^1^, Hong Shen^1^, Mengjiao Nan^1^, Chang Liu^1^, Wenxiang Zhang^1,*^, Siyu Chen^1,*^

^1^State Key Laboratory of Natural Medicines and School of Life Science and Technology, China Pharmaceutical University, Nanjing 211198, China.

***For Correspondence:**

siyuchen@cpu.edu.cn (SYC);

wenxiangzhang@cpu.edu.cn (WXZ);

^†^These authors contributed equally to this work

**Supplementary materials and methods**

**mRNA and protein expression analysis**

Total RNA was isolated using Trizol reagent (Vazyme, Nanjing, China), reverse transcribed, and analyzed by qPCR using SYBR Green (Vazyme, Nanjing, China) and the LightCycler^®^ 480 System (Roche, Basal, Switzerland). The primers for mouse 36B4 were included for normalization of gene expression in mouse liver, spleen, bone marrow and PHs. In addition, the primers for mouse 18S rRNA were included for normalization of gene expression mouse BMDMs. A complete list of PCR primers is shown in Table S1. For protein expression analysis, tissues were homogenized, and BMDMs were lysed in RIPA buffer. Equal amounts of protein were loaded and separated by 10% SDS-PAGE, then transferred onto polyvinylidene difluoride membranes (Millipore, Bedford, MA, USA). The membranes were incubated overnight with appropriate primary antibodies and bound antibodies were then visualized using HRP-conjugated secondary antibodies. A quantitative analysis was performed by using NIH ImageJ 1.32j software. The antibodies against TNF-α (Cat. No. A22227, 1:1000 dilution), IL-6 (Cat. No. A22222, 1:1000 dilution), IL-1β (Cat. No. A22257, 1:1000 dilution), iNOS (Cat. No. A3774, 1:1000 dilution), β-Actin (Cat. No. AC038, 1:1000 dilution), ARG1 (Cat. No. A25808, 1:1000 dilution), JNK1/2/3 (Cat. No. A4867, 1:1000 dilution), p-JNK1/2/3 (AP0631, 1:1000 dilution) were purchased from Abclonal (Nanjing, Jiangsu, China), CD206 (Cat. No. 81525-1-RR; 1:1000 dilution) were purchased from Proteintech (Wuhan, China) and Angptl8 (Cat. No. **MA5-41550**, 1:1000 dilution) were purchased from Themofisher (Waltham, [MA](https://baike.baidu.com/item/MA/9141942?fromModule=lemma_inlink), USA).

**Enzyme-linked immunosorbent assay**

The serum levels of Angptl8 and the cytokines TNF-α, IL-6, IFN-γ were determined by using commercial kits (Ciobo BIO, Shanghai, China for Angptl8; Neobioscience, Beijing, China for TNF-α, IL-6 and IFN-γ). Briefly, serum or cell culture supernatants were collected and analyzed according to the manufacturer’s recommendations.

**Serological analysis**

Blood samples were collected in non-heparinized tubes and centrifuged at 4000 rpm for 10 min at 4 °C. The serum levels of ALT, AST, BUN, creatinine, ANP and BNP were determined spectrophotometrically using commercial kits (Jiancheng Institute of Biotechnology, Nanjing, Jiangsu, China).

**H&E staining**

For H&E staining, fresh samples were fixed in a 4% paraformaldehyde (PFA) solution for 24 h *in situ*, processed for paraffin embedding, and cut into 5μm transverse sections for routine H&E staining. The slides were scanned using a Pannoramic Flash 250 scanner (Perkin Elmer, Waltham, MA, USA) and viewed using the Pannoramic viewer software program (3D Histech, Waltham, MA, USA).

**TUNEL assay**

The TUNEL method was used to detect the effects of Angptl8 deficiency on LPS-induced liver damage. Liver sections were fixed with 4% PFA, and stained with TUNEL Bright Green Apoptosis Detection Kit (Vazyme Biotechnology, China) according to the manufacturer’s instructions. The sections were photographed with a Nikon microscope (ECLIPSE, Ts2R-FL, Tokyo, Japan).

**Complete blood count (CBC) analysis**

Analysis of white blood cell composition and proportion (WBC, Neu#, Mon#, Lym#, Bas#, Eos#) was examined using a Fully Auto Hematology Analyzer (BC 2800-Vet, Mindray, Shenzhen, China).

**Immunofluorescence analysis**

Cells were fixed with 4% paraformaldehyde at room temperature for 30 min and then permeabilized with 0.1% Triton X-100 for 5 min. A blocking step was performed with 1% bovine serum albumin (BSA) for 1 h. Subsequently, the cells were incubated with the primary antibodies overnight at 4 °C. After washing, the cells were incubated with appropriate fluorescent secondary antibodies for 1 h. After three times of washes, the cells were incubated with DAPI (5 μg/mL) at room temperature for 5 min and observed under a fluorescence microscope (ECLIPSE, Ts2R-FL, Tokyo, Japan). Antibodies used in this study included PE anti-mouse CD86 (Biolegend, Cat. No. 105008, 1:100 dilution), anti-CD206 Rab (Proteintech, 81525-1-RR, 1:100 dilution), CoraLite488-conjugated Goat Anti-Rabbit IgG (Proteintech, SA00013-2, 1:100 dilution) and Alexa Fluor® 647 Conjugate Anti-rat IgG (Cell Signaling, 4418, 1:100 dilution).

**Supplementary figures and legends**

**

**

**Fig. S1. The quantitative analysis of** **protein expression of Angptl8 in mice treated with LPS, referred to Fig. 1.** (A) The quantitative analysis of protein expression of Angptl8 in mouse primary hepatocytes treated with indicated doses and time-points of LPS. (B) The quantitative analysis of protein expression of Angptl8 in mouse BMDMs treated with indicated doses and time-points of LPS. ^*^*P* < 0.05 and ^**^*P* < 0.01 *v.s*. No stimulus group, one-way ANOVA followed by Bonferroni’s *posthoc* test, n = 3. (C) The relative mRNA expression of *Angptl8* in the mouse bone marrow. ^**^*P* < 0.01 *v.s*. Saline group, one-way ANOVA followed by Bonferroni’s *posthoc* test, n = 6. All values are presented as the mean ± SD.

**
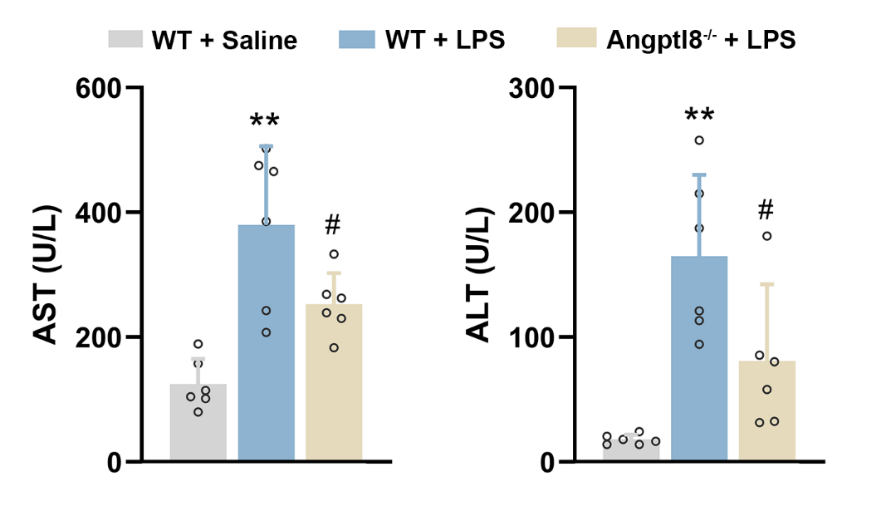
**

**Fig. S2. The mouse serum levels of AST and ALT, referred to Fig. 2.** Serum AST and ALT levels in mice corresponding to Fig. 2. ***P* < 0.01 *v.s.* WT+Saline group, ^#^*P* < 0.05 *v.s.* WT+LPS group, one-way ANOVA followed by Bonferroni’s posthoc test, n = 6. All values are presented as the mean ± SD.

**
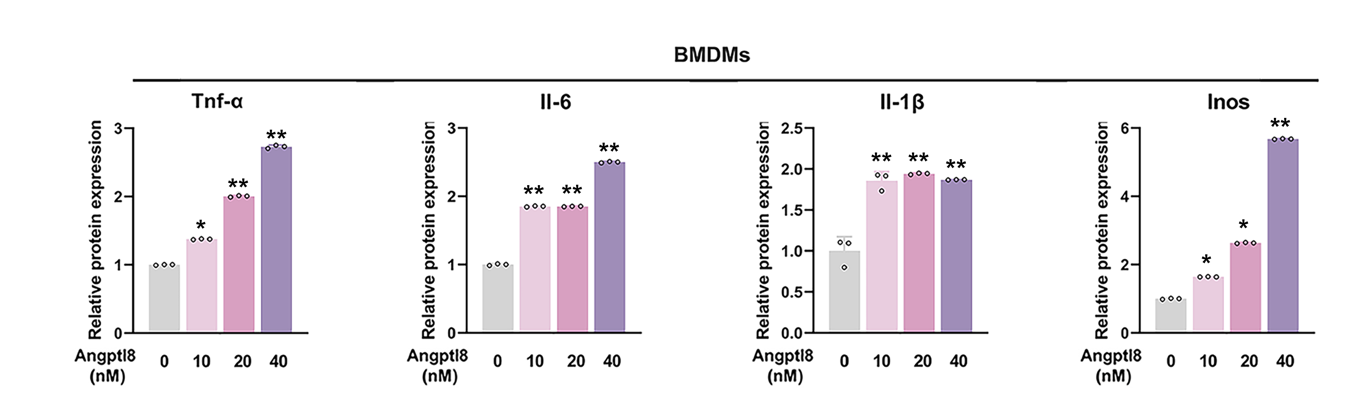
**

**Fig. S3. The quantitative analysis of protein expression of pro-inflammatory factors in BMDMs, referred to Fig. 3H.** The quantitative analysis of protein expression of Tnf-α, Il-6, Il-1β and Inos in BMDMs treated with recombinant Angptl8 (0, 10, 20, 40 nM) for 24 h. ^*^*P* < 0.05 and ^**^*P* < 0.01 *v.s*. No stimulus group, one-way ANOVA followed by Bonferroni’s *posthoc* test, n = 3. All values are presented as the mean ± SD.

**
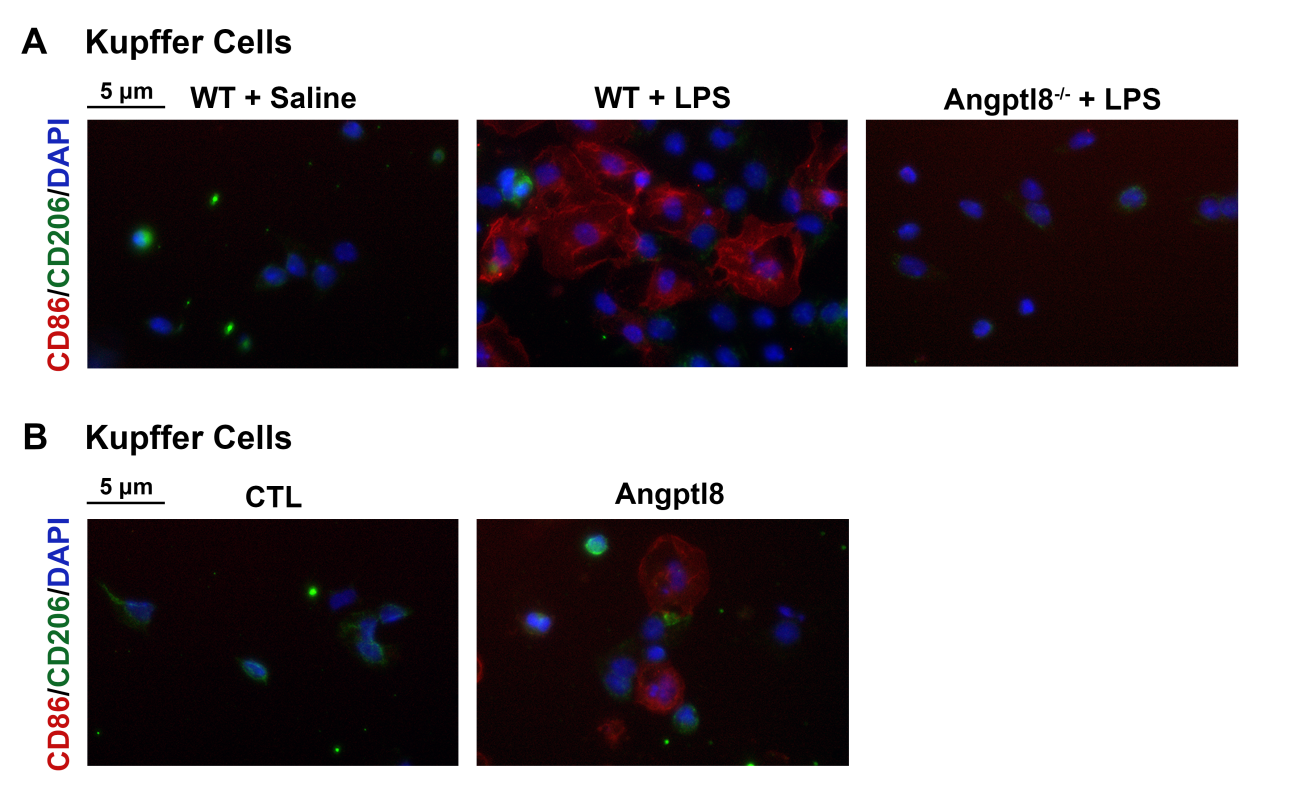
**

**Fig. S4. Immunofluorescence analysis of the effect of Angptl8 treatment on the polarization of liver Kupffer cells, referred to Fig. 3.** (A) Immunofluorescence detection of CD86 (M1 marker, red) and CD206 (M2 marker, green) expression in Kupffer cells isolated from Angptl8^-/-^ and WT mice following LPS stimulation (100 ng/mL, 24h). (B) Immunofluorescence analysis of CD86 and CD206 expression in Kupffer cells isolated from WT mice and treated with recombinant Angptl8. DAPI (blue) was used for nuclear staining. Scale bar: 5 μm.

**
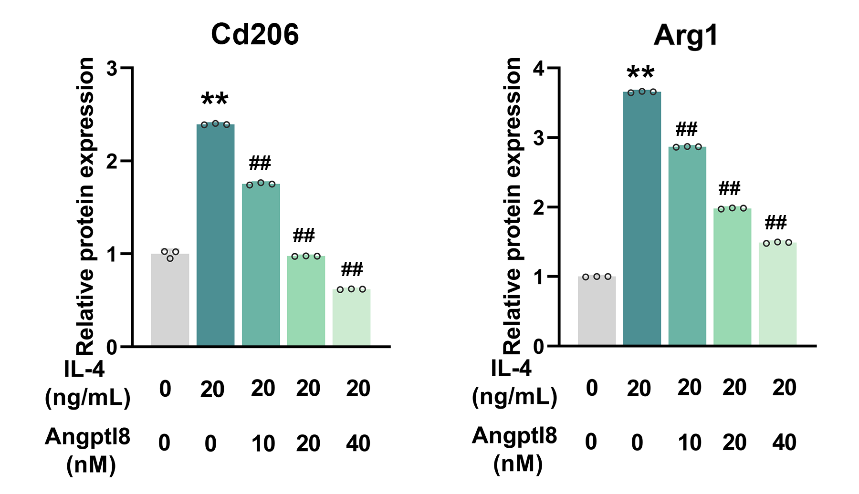
**

**Fig. S5. The quantitative analysis of protein expression of M2-type biomarkers in BMDMs, referred to Fig. 4G.** The quantitative analysis of protein expression of Cd206 and Arg1 in BMDMs treated with IL-4 and recombinant Angptl8. ^*^*P* < 0.05 and ^**^*P* < 0.01 *v.s*. No stimulus group, ^##^*P* < 0.01 *v.s.* IL-4 group, one-way ANOVA followed by Bonferroni’s *posthoc* test, n = 3. All values are presented as the mean ± SD.

**
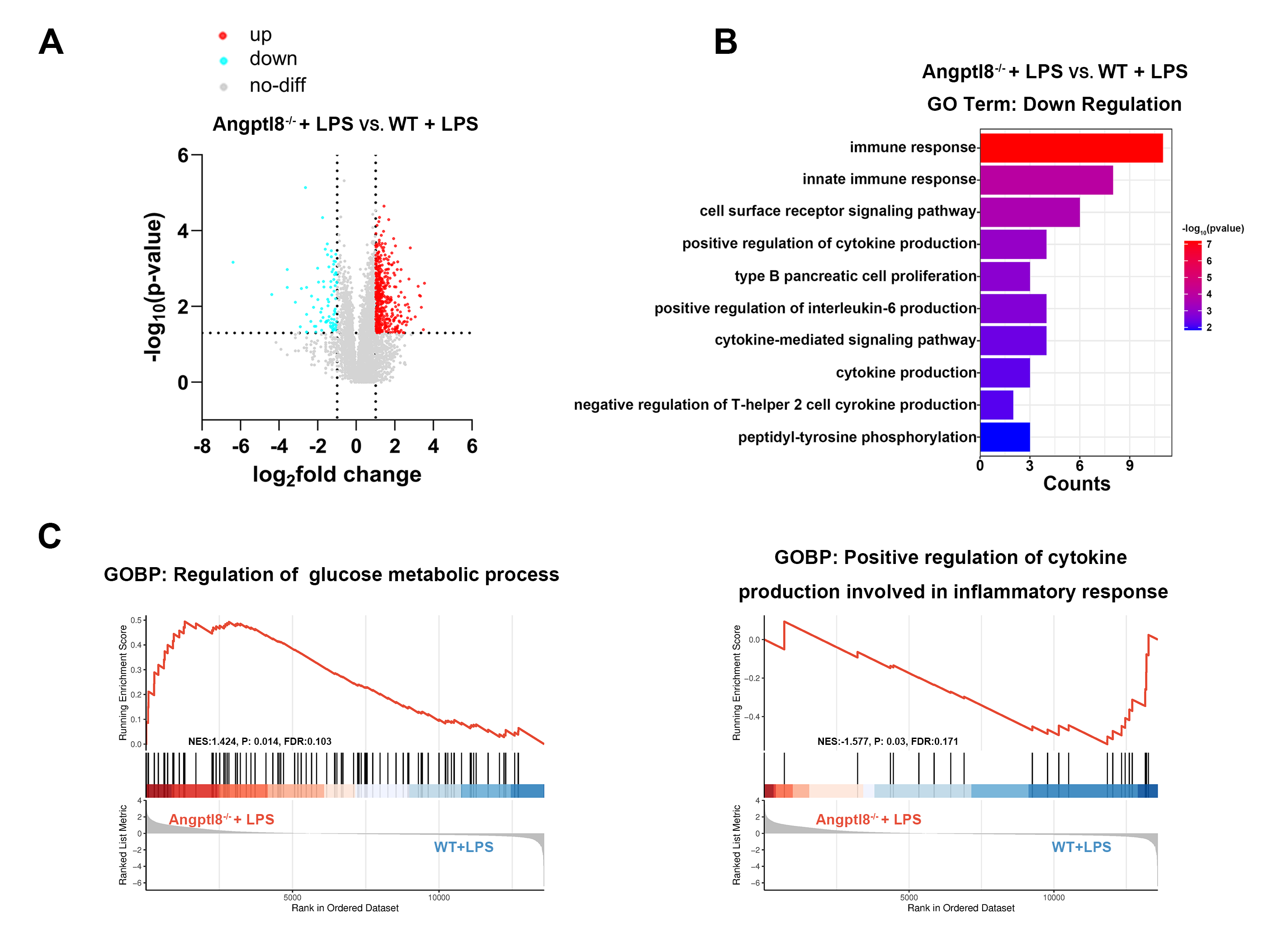
**

**Fig. S6. High-throughput RNA sequencing in the LPS-treated BMDMs from either Angptl8^-/-^ or WT mice, referred to Fig. 5.** (A) Volcano plot showing differentially expressed genes (DEG) in BMDMs between Angptl8^-/-^ + LPS and WT+LPS group. The up-regulated genes (red) with log_2__fold change > 1, and the down-regulated genes (blue) with log_2__fold change < -1, and with *P* < 0.05. (B) GO enrichment analysis of down regulations for key targets. (C) GSEA of key pathways.

**
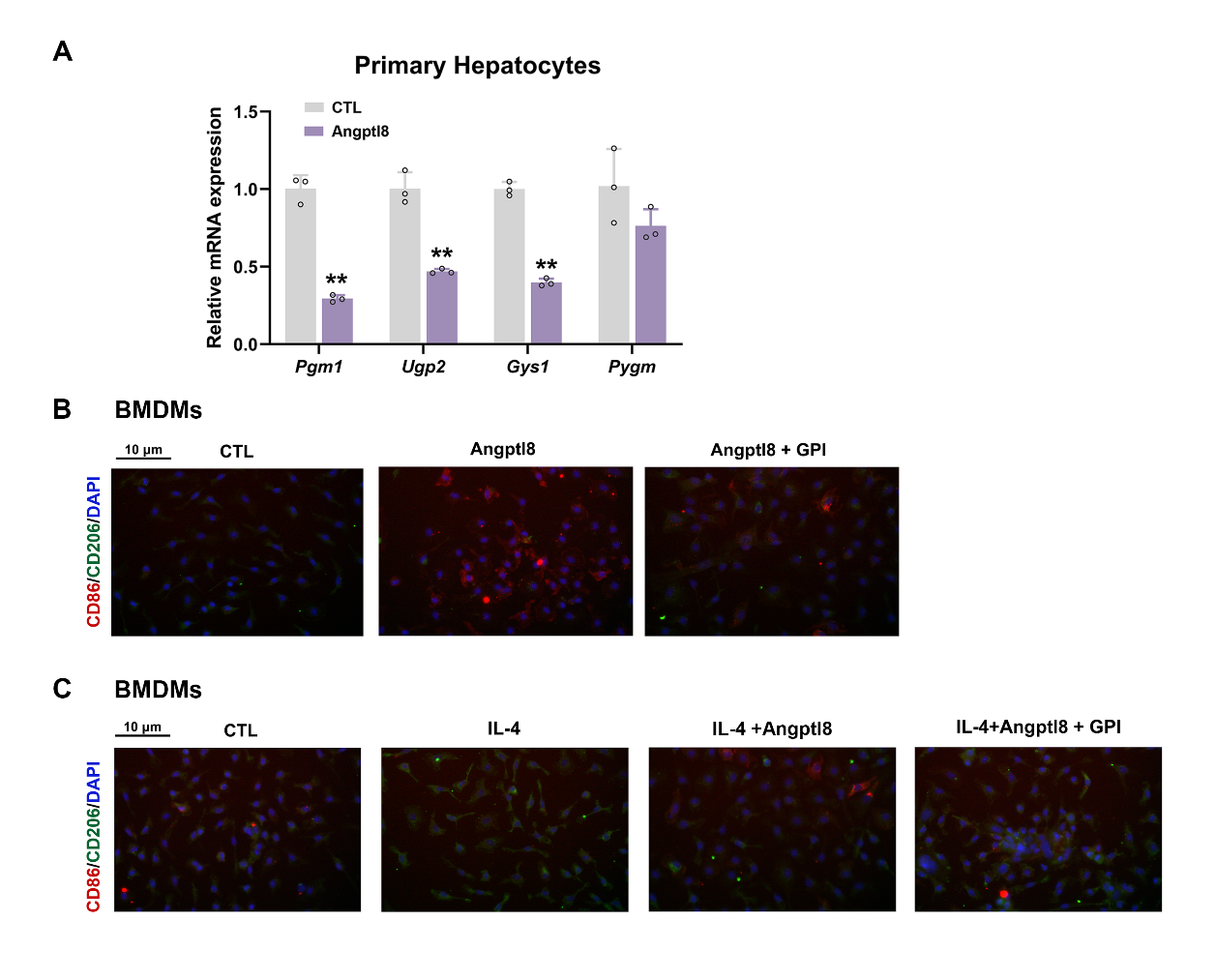
**

**Fig. S7. Angptl8 modulates macrophage polarization in BMDMs through regulation of glycogen metabolism, referred to Fig. 5.** (A) The relative mRNA expression of *Pgm1*, *Ugp2* and *Gys1* and *Pygm* in mouse primary hepatocytes treated with recombinant Angptl8. ^**^*P* < 0.01 *v.s*. No stimulus group, one-way ANOVA followed by Bonferroni’s *posthoc* test, n = 3. All values are presented as the mean ± SD. (B) Immunofluorescence analysis of CD86 (M1 marker, red) and CD206 (M2 marker, green) expression in BMDMs treated with recombinant Angptl8 and GPI. (C) Immunofluorescence detection of CD86 and CD206 expression in BMDMs treated with IL-4, recombinant Angptl8 and GPI. DAPI (blue) was used for nuclear staining. Scale bar: 10 μm.


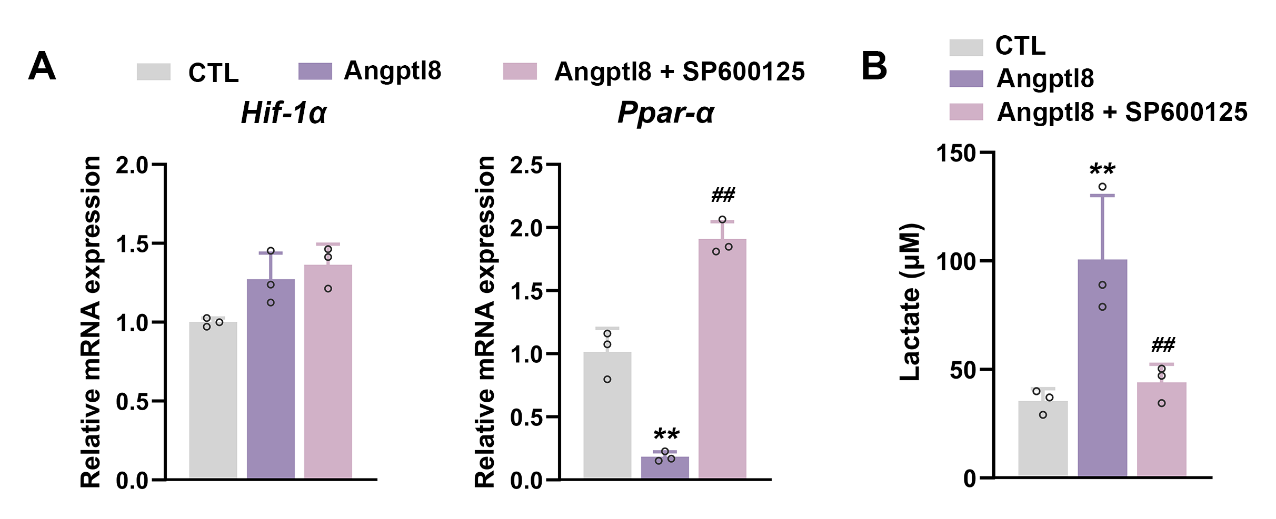


**Fig. S8.** **Phosphorylated JNK mediates Angptl8-induced activation of glycogen metabolism in macrophages, referred to Fig. 6.** (A) The relative mRNA expression of *Hif-1α* and *Ppar-α* in mouse primary hepatocytes treated with recombinant Angptl8 and SP600125. (B) lactate acid levels in mouse BMDMs treated with recombinant Angptl8 and SP600125. ^**^*P* < 0.01 *v.s*. No stimulus group, ^##^*P* < 0.01 *v.s.* Angptl8 group, one-way ANOVA followed by Bonferroni’s *posthoc* test, n = 3. All values are presented as the mean ± SD.

**
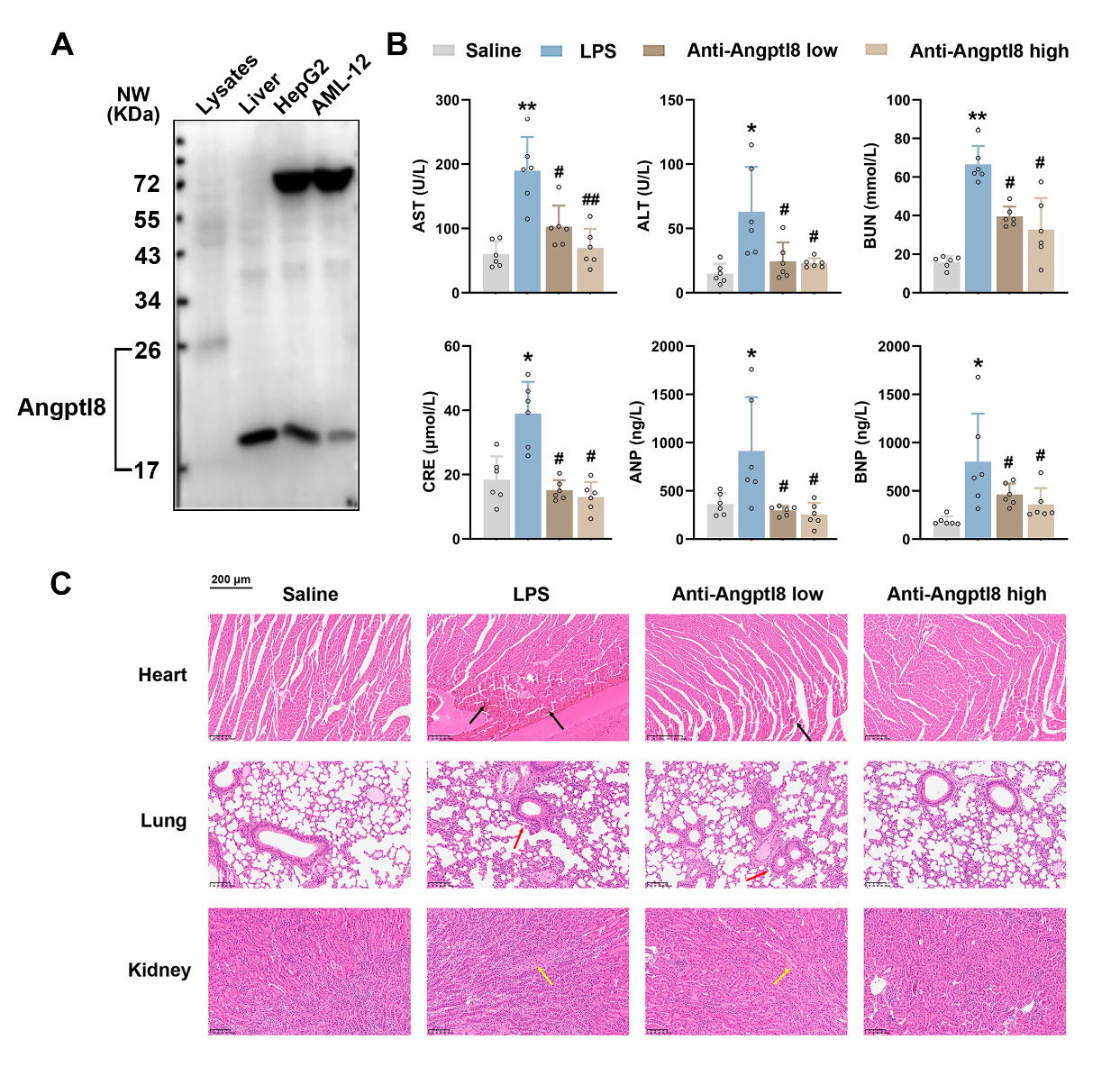
**

**Fig. S9. Biosafety assessment of neutralizing Angptl8 antibodies, referred to Fig. 7.** (A) protein expression of Angptl8 in Lysates, liver, HepG2 and AML-12 treated with neutralizing anti-Angptl8 antibody. (B) The serum levels of AST, ALT, creatinine and BUN, ANP and BNP in mice corresponding to Fig. 7. ^*^*P* < 0.05 and ^**^*P* < 0.01 *v.s*. Saline group, ^#^*P* < 0.05 and ^##^*P* < 0.01 *v.s*. LPS group, one-way ANOVA followed by Bonferroni’s *posthoc* test, n = 6. All values are presented as the mean ± SD. (C) Representative images of heart, lung and kidney by H&E staining analysis.

**Table S1. Lists of primer sequences for RT-qPCR analysis.**

| **Mouse Genes** | **Forward Primer Sequences (5’-3’)** | **Reverse Primer Sequences (5’-3’)** |
| --- | --- | --- |
| *36B4* | GAAACTGCTGCCTCACATCCG | GCTGGCACAGTGACCTCACACG |
| *18s rRNA* | AGTCCCTGCCCTTTGTACACA | CGATCCGAGGGCCTCACTA |
| *Angptl8* | CTCAATGGCGTGTACAGAGC | TCGAAGGTGTAAAGCGTCCT |
| *Tnf-α* | CAGGCGGTGCCTATGTCTC | CGATCACCCCGAAGTTCAGTAG |
| *Il-6* | CTGCAAGAGACTTCCATCCAG | AGTGGTATAGACAGGTCTGTTGG |
| *Il-1β* | AGTTGACGGACCCCAAAAG | AGCTGGATGCTCTCATCAGG |
| *Inos* | GGAGTGACGGCAAACATGACT | TCGATGCACAACTGGGTGAAC |
| *Cd206* | CTCTGTTCAGCTATTGGACGC | TGGCACTCCCAAACATAATTTGA |
| *Arg1* | TGTCCCTAATGACAGCTCCTT | GCATCCACCCAAATGACACAT |
| *Ym1* | CAGGTCTGGCAATTCTTCTGAA | GTCTTGCTCATGTGTGTAAGTGA |
| *Fizz1* | CCAATCCAGCTAACTATCCCTCC | ACCCAGTAGCAGTCATCCCA |
| *Gys1* | CACAGAACGGTTGTCGGACTTG | AGGTGAAGTGGTCTGGAAAGGC |
| *Pgm1* | AGCCAATGACCCAGATGCTGAC | TCCAGGAAGTGAAGAGCCACCA |
| *Ugp2* | CTGATGAACCCACCCAATGGGA | GAGCGATTTCCACCAGTCTCAG |
| *Pygm* | ATGGCACACCTGTGCATTGCTG | CGAGGAGTGATGCCATTGGTCT |
| *Hif-1α* | TCTCGGCGAAGCAAAGAGTC | AGCCATCTAGGGCTTTCAGATAA |
| *Ppar-α* | AACATCGAGTGTCGAATATGTGG | CCGAATAGTTCGCCGAAAGAA |
