# Supplementary figures and images for: Angiopoietin-like protein 8 orchestrates macrophage glycogen metabolism and polarization via the JNK signaling pathway in cytokine storm syndrome
Source: Cell Biosci. 2025 Oct 15;15:140. doi: 10.1186/s13578-025-01487-7 (PMC12522911; doi:10.1186/s13578-025-01487-7)

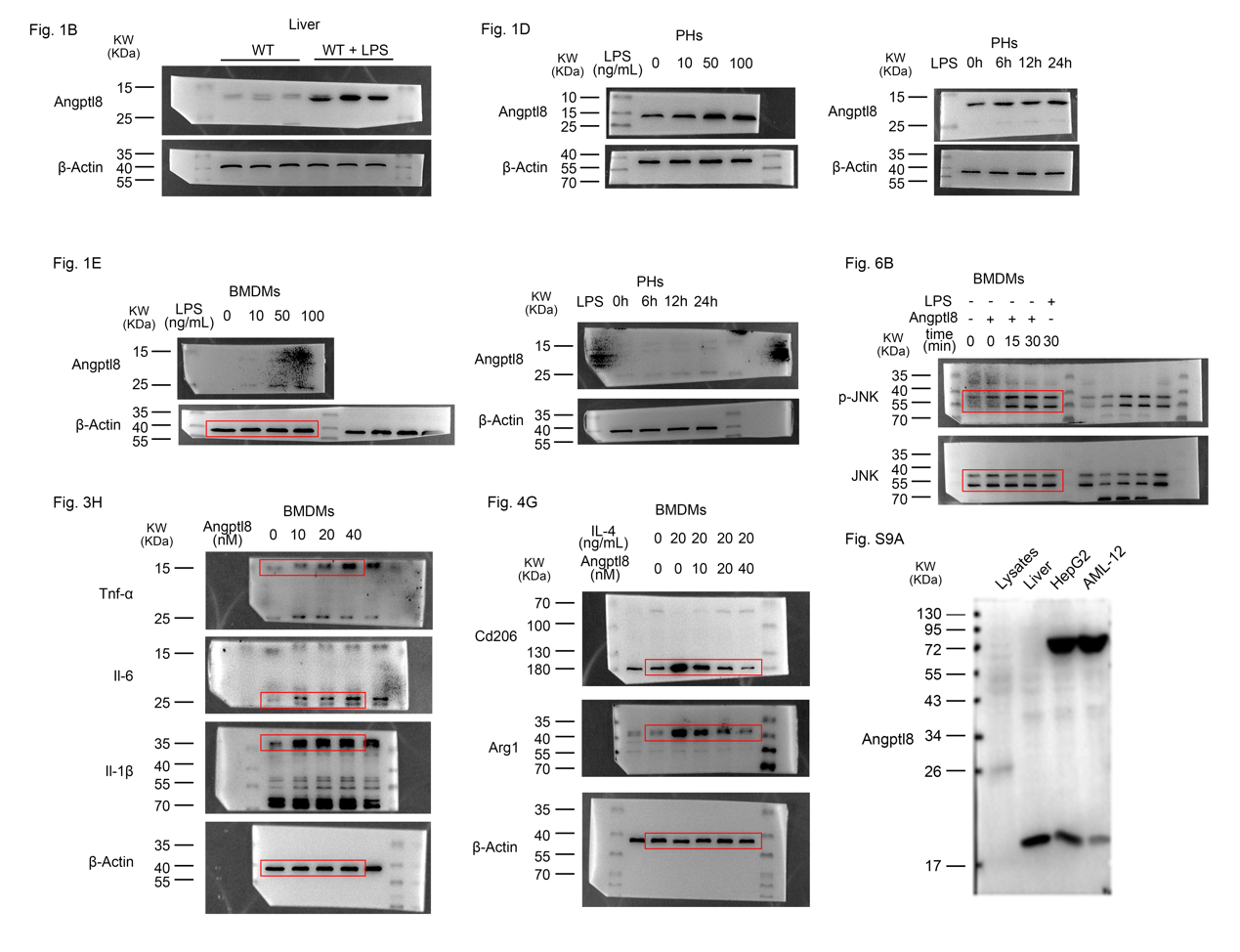

Supplement: Supplementary file 2 — Supplementary Material 2 [file 13578_2025_1487_MOESM2_ESM.docx]
